# Impact of ROS-Induced Damage of TCA Cycle Enzymes on Metabolism and Virulence of *Salmonella enterica* serovar Typhimurium

**DOI:** 10.3389/fmicb.2019.00762

**Published:** 2019-04-24

**Authors:** Janina Noster, Marcus Persicke, Tzu-Chiao Chao, Lena Krone, Bianca Heppner, Michael Hensel, Nicole Hansmeier

**Affiliations:** ^1^Abteilung Mikrobiologie, Universität Osnabrück, Osnabrück, Germany; ^2^Microbial Genomics and Biotechnology, Center for Biotechnology, Bielefeld University, Bielefeld, Germany; ^3^Institute of Environmental Change and Society, University of Regina, Regina, SK, Canada; ^4^Luther College, University of Regina, Regina, SK, Canada

**Keywords:** metabolomics, oxidative stress, iron–sulfur cluster damage, aconitase, superoxide dismutase

## Abstract

*Salmonella enterica* serovar Typhimurium (STM) is exposed to reactive oxygen species (ROS) originating from aerobic respiration, antibiotic treatment, and the oxidative burst occurring inside the *Salmonella*-containing vacuole (SCV) within host cells. ROS damage cellular compounds, thereby impairing bacterial viability and inducing cell death. Proteins containing iron–sulfur (Fe–S) clusters are particularly sensitive and become non-functional upon oxidation. Comprising five enzymes with Fe–S clusters, the TCA cycle is a pathway most sensitive toward ROS. To test the impact of ROS-mediated metabolic perturbations on bacterial physiology, we analyzed the proteomic and metabolic profile of STM deficient in both cytosolic superoxide dismutases (Δ*sodAB*). Incapable of detoxifying superoxide anions (SOA), endogenously generated SOA accumulate during growth. Δ*sodAB* showed reduced abundance of aconitases, leading to a metabolic profile similar to that of an aconitase-deficient strain (Δ*acnAB*). Furthermore, we determined a decreased expression of *acnA* in STM Δ*sodAB*. While intracellular proliferation in RAW264.7 macrophages and survival of methyl viologen treatment were not reduced for STM Δ*acnAB*, proteomic profiling revealed enhanced stress response. We conclude that ROS-mediated reduced expression and damage of aconitase does not impair bacterial viability or virulence, but might increase ROS amounts in STM, which reinforces the bactericidal effects of antibiotic treatment and immune responses of the host.

## Introduction

Pathogenic bacteria are continuously exposed to reactive oxygen species (ROS), which arise from three major sources. (i) During aerobic respiration, intrinsic ROS formation occurs by the electron transport chain (Messner and Imlay, 1999). (ii) ROS production is one of the antimicrobial mechanisms of bacterial clearance by professional phagocytes (Aussel et al., 2011). (iii) Additionally, recent studies demonstrated increased concentration of intrinsic ROS induced by antibiotic treatment (Kohanski et al., 2007; Belenky et al., 2015). In all three cases, superoxide anions (SOA) are generated, which dismutate to hydrogen peroxide (HPO) (Craig and Slauch, 2009). In contrast to HPO, SOA are negatively charged, thus unable to cross membranes. This gives rise to an ongoing debate of the primary targets in bacterial cells leading to clearance by immune cells (De Groote et al., 1997; Mumbengegwi et al., 2008; Craig and Slauch, 2009; Slauch, 2011). During early steps of phagosome maturation, NADPH oxidase is recruited to the phagosome membrane and increases SOA concentration within the compartment (Kamen et al., 2008; Craig and Slauch, 2009). Due to the acidic pH in the phagosome, a fraction of SOA is protonated to perhydroxyl radical (PHO), capable to transverse membranes and to access the bacterial cytosol (Korshunov and Imlay, 2002). All three kinds of ROS attack sensitive bacterial macromolecules. Iron–sulfur (Fe–S) clusters become oxidized and iron is liberated, reacting in Fenton–Haber–Weiss reactions with SOA and HPO, respectively, to hydroxyl radicals (HR), the most harmful form of ROS (Craig and Slauch, 2009). Being much less restricted in targets, HR damage various macromolecules within the bacterial cell, leading to reduced growth or even cell death (Kobayashi et al., 2014).

Bacteria such as the facultative intracellular pathogen *Salmonella enterica* serovar Typhimurium (STM), the causative agent of food-borne gastroenteritis, possess several oxidative stress response systems. Cytosolic superoxide dismutases SodA and SodB convert SOA to HPO, which is further detoxified by catalases (Blanchard et al., 2007). In the case the capacity of constitutively expressed enzymes is overrun, SoxRS and OxyRS regulons are induced by SOA and HPO, respectively (Christman et al., 1989; Pomposiello and Demple, 2000) (reviewed in Pomposiello and Demple, 2001). Enhanced expression of regulon members prevent further increase of ROS concentrations (i.e., *sodA, ahpC, ahpF, katG, katE, katN, tpx, gor, grxA, zwf*), or protect and repair structures easily damaged by ROS (i.e., *dps, nfo, zwf*) (Zheng et al., 1999; Pomposiello et al., 2001; Hebrard et al., 2009). By increased abundance of glucose-6-phosphate-1-dehydrogenase (G6PD, Zwf), the metabolic flux can be redirected toward the pentose phosphate pathway (PPP), which rises the pool of NADPH, important for the regeneration of thioredoxins and glutaredoxins (Seo et al., 2015; Christodoulou et al., 2018). To maintain TCA cycle activity during oxidative stress, STM induces expression of *acnA* and *fumC* in a SoxRS-dependent manner, and AcnA and FumC are isoforms of aconitase and fumarase less sensitive to oxidation (Gu and Imlay, 2011).

Even though STM induces stress response regulons, the capacity of the bacterial cell to cope with increasing ROS levels is limited. In case of continuous oxidative stress, important macromolecules are damaged with fatal consequences for bacterial physiology. Of highest impact is the oxidation of DNA bases, especially guanine, leading to mutagenic modifications, strand breaks and cell death if not repaired immediately (Lloyd et al., 1998; Fang, 2011). However, also inactivation of metabolic enzymes can have far-reaching consequences: continuous exposition to SOA was mentioned to impair amino acid biosynthesis and thus induce auxotrophies for branched-chain, sulfur-containing, and aromatic amino acids (Benov and Fridovich, 1999). Further studies focused on the impact of oxidative stress on the bacterial metabolism and reported decreased activity or abundance of enzymes harboring Fe-S clusters and glycolytic enzymes. The outcome of those studies varied, as they are strongly influenced by molecular type, intensity and duration of ROS stress (paraquat, HPO, etc.), the growth medium (rich medium vs. defined minimal media), and culture technique (static culture vs. chemostat culture). Furthermore, results are biased by the growth phase during stress exposure, the choice of bacterial species used and the analyses technique, i.e., metabolic flux analysis, proteomics, transcriptomics, metabolomics (Kim et al., 2010; Fu et al., 2017). Nonetheless, an influence on the TCA cycle was demonstrated in all studies, yet to different extent. Harboring five enzymes with Fe-S clusters, the TCA cycle represents one of the most sensitive pathways with respect to ROS attacks (Jordan et al., 1999; Tseng et al., 2001; Cheng et al., 2006).

*Salmonella enterica* serovar Typhimurium experiences continuous oxidative stress during intracellular presence in the phagosome, in the specific pathogen-containing compartment (*Salmonella*-containing vacuole, SCV) (van der Heijden et al., 2015), or during long-term treatment with antibiotics (Kohanski et al., 2007; Kaiser et al., 2014; Belenky et al., 2015). ROS accumulation inside bacteria leads to severe damage of cellular compounds (Rui et al., 2010). The impact of oxidative stress on bacterial metabolism was often discussed (Rui et al., 2010; Shen et al., 2013; Drazic et al., 2015), but how ROS-induced perturbations of metabolic pathways alone lead to reduced viability, was not studied.

In order to examine effects of continuous oxidative stress, we performed metabolomics and proteomics of a mutant strain defective in both cytosolic superoxide dismutases (STM Δ*sodAB*). We demonstrate that under these conditions metabolic perturbations of STM Δ*sodAB* were to a great extent influenced by reduced abundance of aconitases, leading to a metabolic profile strikingly similar to that one of STM with deleted aconitases (STM Δ*acnAB*). Additionally, real-time experiments revealed reduced expression of *acnA* in STM Δ*sodAB*. Although aconitase deletion did not decrease bacterial viability and virulence in our experimental setup, proteomic profiling of the mutant strain indicated an increased stress response. Thus, impairment of aconitases under oxidative stress might increase ROS exposure in STM.

## Results

### A *sodAB* Deletion Strain, Unable to Neutralize SOA, Is Exposed to High Levels of Oxidative Stress

While most previous studies analyzed the effects of ROS in ‘pulse-chase’ experiments with short-term ROS exposure (Wang et al., 2009; Allen and Griffiths, 2012; Drazic et al., 2015; Fu et al., 2017), we elucidated the consequences of long-term oxidative stress on STM metabolism. By deletion of the cytosolic superoxide dismutases SodA and SodB, we abolish the ability of STM to neutralize cytosolic SOA and therefore expect a higher exposure to oxidative stress compared to the WT (Gort and Imlay, 1998). A known phenotype of *Escherichia coli* with defects in both cytosolic superoxide dismutases is an auxotrophy for branched, sulfur-containing and aromatic amino acids (Benov and Fridovich, 1999). Indeed, Δ*sodAB* STM was unable to grow in minimal medium without amino acid supplementation, indicating SOA-mediated damage of enzymes required for amino acid biosynthesis (see Supplementary Figure S1 and aerobic, and Supplementary Figure S2 for anaerobic growth).

We used comparative proteome analyses to estimate oxidative stress levels for STM WT and STM Δ*sodAB*. We cultured STM WT and STM Δ*sodAB* in rich LB broth, which ensured similar growth kinetics of both STM strains (see Supplementary Figure S1A) and analyzed their proteomes by LC-MS^E^. We observed increased abundance of six stress-related proteins in STM Δ*sodAB* compared to WT (see Table 1 and Supplementary Table S1), i.e., Ssb (single-stranded DNA binding protein: 3.11-fold), SodC2 (superoxide dismutase; 6.12-fold), TreA (periplasmic trehalase: 6.32-fold), UspE/G (universal stress proteins E and G: 7.58-fold and 10.45-fold, respectively), and ElaB (putative inner membrane protein: 12.56-fold). All differentially abundant proteins in STM Δ*sodAB* are known to be important for oxidative stress resistance and DNA damage repair in STM or *E. coli* (Nachin et al., 2005; Ammendola et al., 2008; Huang and Huang, 2014; Tidhar et al., 2015; Guo et al., 2017). Thus, in line with previous studies (Baez and Shiloach, 2013) we can conclude that the inability of STM Δ*sodAB* to neutralize endogenous SOA leads to increased oxidative stress, which allows us to mimic long-term oxidative stress exposure.

**Table 1:** Differentially abundant stress-response proteins of STM Δ*sodAB* in comparison to WT^∗^.

	*Gene product*	*Protein description*	*Ratio Δ*sodAB*/WT*
Less abundant	KatE	Catalase	0.35
in STM	TrxB	Thioredoxin reductase	0.47
*Δ*sodAB**	HtpG	Chaperone protein	0.48
	YgaM	Putative inner membrane protein	0.81
More abundant in STM	Ssb	Single-stranded DNA-binding protein 1	3.11
*Δ*sodAB**	SodC2	Superoxide dismutase	6.12
	TreA	Periplasmic trehelase	6.32
	UspE	Universal stress protein	7.58
	UspG	Universal stress protein	10.45
	ElaB	Putative inner membrane protein	12.56

*^∗^Listed are proteins classified in category ‘stress response’ according to Gene Ontology (biological process). Only proteins with significantly different amounts are depicted (Student’s t-test, p < 0.05).*

### Long-Term Oxidative Stress Influences Enzyme Abundance in Primary Metabolic Pathways of STM

**FIGURE 1:**
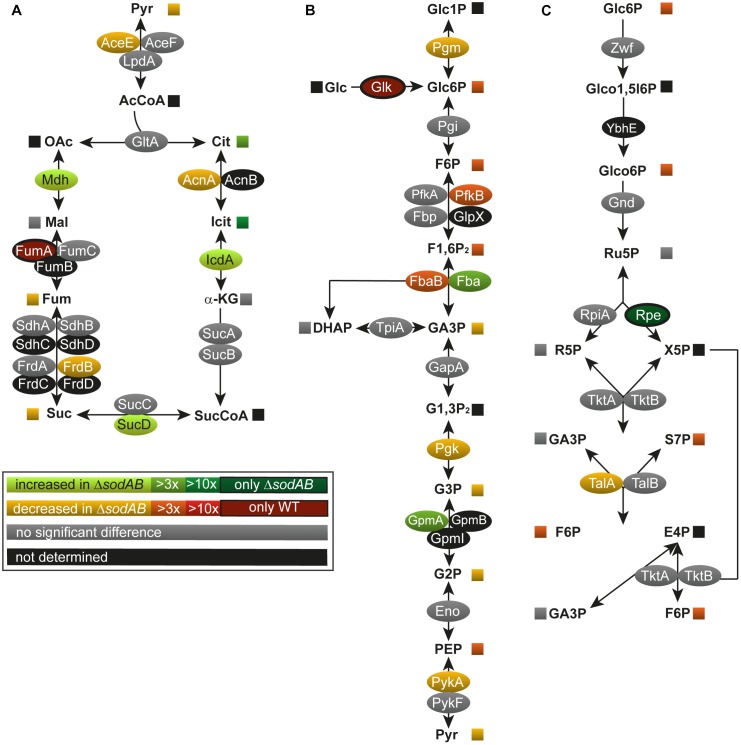
Deletion of both cytosolic superoxide dismutases impacts metabolite concentrations and enzyme abundance of TCA cycle **(A)**, glycolysis **(B)**, and pentose phosphate pathway **(C)**. STM WT and STM Δ*sodAB* strains were grown aerobically in LB broth for 18.5 h at 37°C, harvested and proteomes or metabolomes were extracted for quantitative profiling using LC-MS^E^ or GC-MS, respectively. Data represent means of at least three biological replicates for metabolomics or proteomics analyses. Data were adjusted for multiple hypothesis testing and only significant differences with *p* < 0.05 or lower are displayed color-encoded. Oval symbols indicate relative changes in enzyme amounts detected for STM Δ*sodAB* compared to WT, whereas squares reflect relative changes in amounts of metabolites. Gray symbols are indifferent in enzyme or metabolite amounts.

To address how long-term oxidative stress provokes metabolic perturbations, we analyzed the primary metabolism of WT and STM Δ*sodAB*. After classification of the identified proteins according to gene ontology (biological process) (Ashburner et al., 2000; The Gene and Ontology Consortium, 2017), a total of 11 proteins related to metabolic pathways were significantly more abundant in STM Δ*sodAB* compared to WT, 27 proteins were less abundant, indicating a reduced metabolic activity of the ROS-sensitive mutant strain. A significant decline was found in the abundance of TCA cycle and TCA cycle-associated proteins (aconitase A, AcnA: 1.86-fold reduction, fumarate reductase B, FrdB: 2.23-fold, and pyruvate dehydrogenase, AceE: 1.88-fold), as well as glycolysis enzymes (phosphoglucomutase, Pgm: 1.43-fold, phosphofructokinase B, PfkB: 4.42-fold, fructose-bisphosphate aldolase, Fba: 3.03-fold, phosphoglycerate kinase, Pgk: 2.32-fold, and pyruvate kinase, PykA: 2.14-fold) in STM Δ*sodAB* compared to WT (Figure 1). Unexpectedly, we detected no changes in the level of fumarase C (FumC), while fumarase A (FumA) was only detected for STM WT.

**FIGURE 2:**
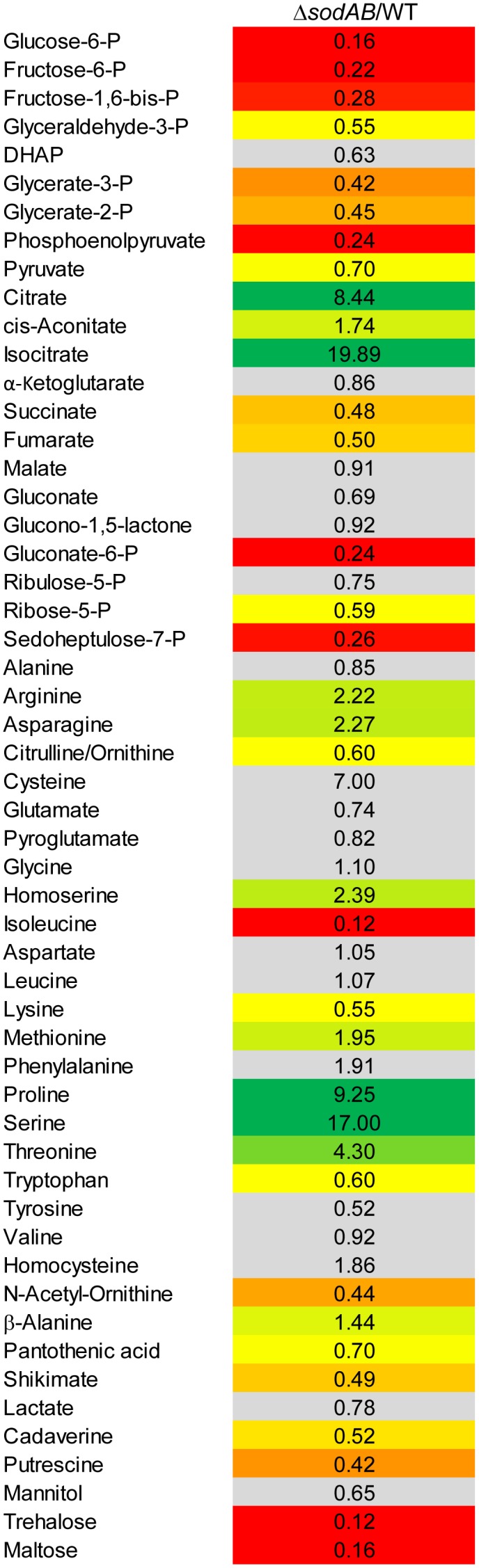
Metabolic profile of *sodAB*-deficient STM compared to WT. STM WT and STM Δ*sodAB* were cultured aerobically in LB broth for 18.5 h at 37°C, before cells were harvested and metabolites for subsequent GC-MS analyses extracted. Metabolite levels were normalized to WT levels and means of four biological replicates are shown. The color code indicates relative metabolite concentration in STM Δ*sodAB* compared to WT (red = decreased; green = increased). Gray color show indifferent metabolite concentrations between STM Δ*sodAB* and WT (Student’s *t*-test, *p* < 0.05).

Among metabolic proteins with enhanced abundance we identified isocitrate dehydrogenase (IcdA: 2.26-fold increase), succinyl-CoA synthetase (SucD: 1.70-fold), malate dehydrogenase (Mdh: 2.56-fold) as well as fructose-bisphosphate aldolase (Fba: 3.29-fold) and phosphoglycerate mutase (GpmA; 4.94-fold) in STM Δ*sodAB*. Furthermore, we detected a decline in the PPP enzymes. Transaldolase A (TalA) was detected in reduced levels in STM Δ*sodAB* compared to WT (2.35-fold reduced) and ribulose-phosphate-3-epimerase (Rpe) was only detected in the superoxide dismutase defective mutant. Our proteomic data were supported by metabolic analyzes of cell lysates by GC-MS measurement (Figure 1, 2 and Supplementary Table S2).

Several key metabolites from CCM (central carbon metabolism) pathways were detected. As expected, overall metabolite levels were found in lower concentrations in STM Δ*sodAB* compared to WT (1.42- to 6.14-fold reduced). Only two metabolites were detected in higher levels in STM Δ*sodAB*, i.e., citrate (8.44-fold increased) and isocitrate (19.9-fold increased). Thus, we can conclude that the inability of STM Δ*sodAB* to neutralize endogenous SOA leads to reduced carbon metabolism.

Further alterations in the metabolic profile of STM Δ*sodAB* compared to WT were observed for amino acid concentrations (see Figure 2 and Supplementary Figure S3). Whereas levels of arginine, asparagine, homoserine and methionine were increased about 2-fold, we detected high accumulations of threonine (4.3-fold), proline (9.25-fold), and serine (17-fold). Proteomic data showed no striking increase of protein abundances of either ABC transporters nor synthesis enzymes of the respective amino acids. Therefore, increased metabolite levels may be reasoned by changed metabolic flux due to modified catalytic activities of amino acid synthesis enzymes induced by continuous oxidative stress. Reduced concentrations were observed for citrulline/ornithine (1.67-fold), lysine (1.82-fold), tryptophan (1.67-fold), and reduction of isoleucine levels was most severe (8.15-fold). The latter two values are indicative for oxidative stress induced amino acid auxotrophies (Gort and Imlay, 1998). Indeed, growth of Δ*sodAB* in minimal medium with glucose as sole carbon source could be restored by supplementation with the respective amino acids after 8 h (OD_600_ 0.086 ± 0.015, and 1.8 ± 0.2, without and with supplementation, respectively), whereas growth of STM WT was only slightly affected (OD_600_ 3.2 ± 0.5 and 4.6 ± 0.1 without and with supplementation, respectively) (see Supplementary Figure S1). To test if tryptophan and isoleucine are limited in spent supernatant of STM Δ*sodAB*, but not WT cultured in LB broth after 18.5 h of growth, mutant strains auxotrophic for isoleucine (Δ*ilvA*) or tryptophan (Δ*trpC*) were generated. STM WT, Δ*sodAB* and both auxotrophic mutant strains were grown o/n in LB broth. Supernatants were sterile filtered and each strain inoculated to an OD_600_ of 0.01 in each spent supernatant (Table 2). Whereas growth in spent supernatant of WT, Δ*ilvA* and Δ*trpC* was strongly reduced (OD_600_ = 0.115–0.125 after 24 h of growth), higher optical densities were achieved in spent supernatant of STM Δ*sodAB* (OD_600_ = 1.4–1.7). These results indicate that isoleucine as well as tryptophan are not completely exhausted in spent supernatant of Δ*sodAB*. Therefore, we assume that uptake rates for the respective amino acids in Δ*sodAB* are not sufficiently effective to cope the increased demand due to induced auxotrophies. Proteomic analyses revealed reduced levels of ArgT, a subunit of the ABC transporter required for uptake of lysine, arginine and ornithine in *Δ*sodAB** compared to WT, whereas one further subunit (HisM) was detected in STM WT only. Thus, reduced lysine and ornithine levels may be decreased as a consequence of reduced uptake. Furthermore, increased flux from ornithine to polyamines was reported to be reduced in *E. coli* exposed to oxidative stress (Tkachenko et al., 2012). However, putrescine levels were decreased in *Δ*sodAB** compared to WT.

**Table 2:** Growth of STM WT, Δ*sodAB* and auxotrophic mutant strains in spent supernatants.

Strain	Supernatant from	o/n culture OD_600_ mean ± standard deviation
WT	WT	0.125 ± 0.007
	Δ*sodAB*	1.700 ± 0.424
	Δ*ilvA*	0.115 ± 0.007
	Δ*trpC*	0.115 ± 0.007
Δ*sodAB*	WT	0.050 ± 0.000
	Δ*sodAB*	0.055 ± 0.007
	Δ*ilvA*	0.040 ± 0.000
	Δ*trpC*	0.045 ± 0.007
Δ*ilvA*	WT	0.125 ± 0.007
	Δ*sodAB*	1.650 ± 0.071
	Δ*ilvA*	0.120 ± 0.000
	Δ*trpC*	0.125 ± 0.021
Δ*trpC*	WT	0.125 ± 0.007
	Δ*sodAB*	1.400 ± 0.424
	Δ*ilvA*	0.115 ± 0.007
	Δ*trpC*	0.115 ± 0.007

### Comparison of the Metabolic Profile of STM Δ*sodAB* to STM Lacking Iron–Sulfur Cluster Containing TCA Cycle Enzymes

The TCA cycle plays a significant role during ROS exposure. On the one hand, the TCA cycle indirectly enhances ROS accumulation by generating reduction equivalents feeding the electron transport chain and its catalytic activity is thought to be enhanced by antibiotic treatment (Kohanski et al., 2007). On the other hand, the pathway itself is highly susceptible to ROS, since five enzymes of this pathway contain Fe-S clusters (Flint et al., 1993) (aconitase A, aconitase B, succinate dehydrogenase subunit B, fumarase A, fumarase C). Indeed, our proteomic data indicated that levels of aconitases and fumarases were reduced in STM Δ*sodAB*. Metabolic data supported these data by the high accumulation of citrate detected. Fumarate was not accumulated in STM Δ*sodAB*, likely due to the unchanged levels of fumarase C and the already reduced fluxes through the TCA cycle due to damages in the upper part of the pathway.

**FIGURE 3:**
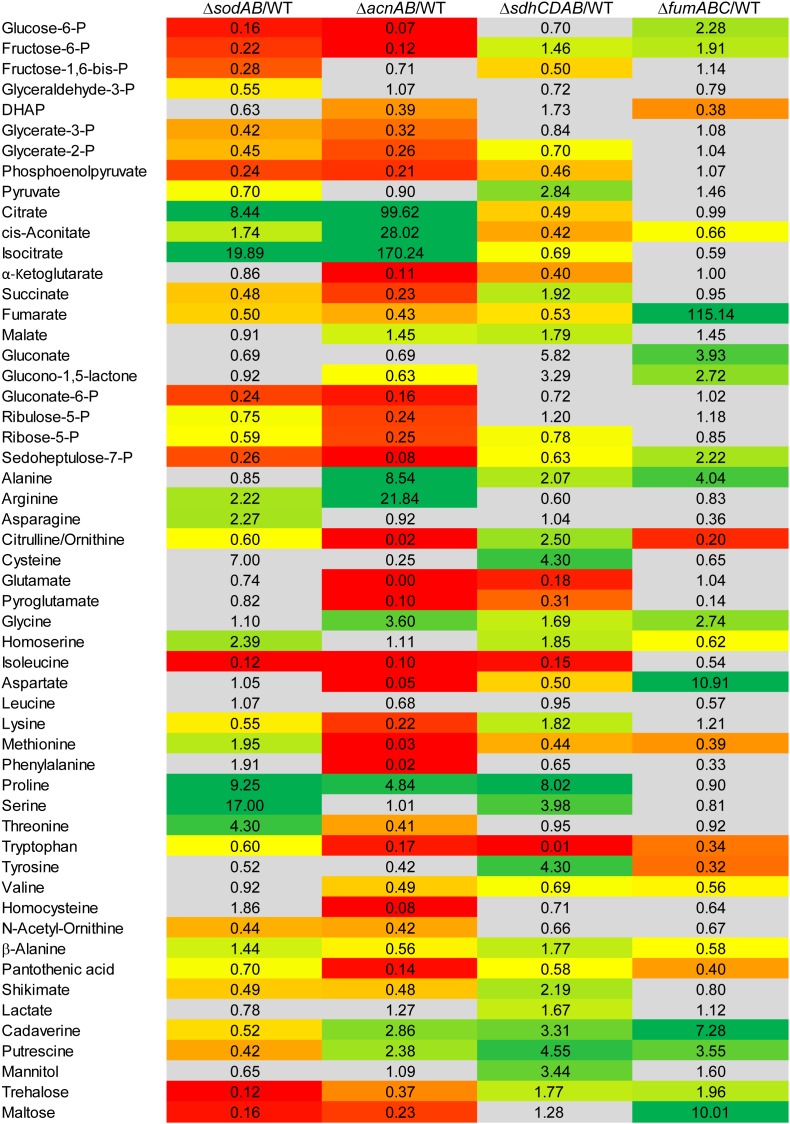
Comparison of the metabolic profiles of STM Δ*sodAB* and mutant strains defective in aconitases or fumarases and succinate dehydrogenase. STM WT and STM Δ*sodAB*, STM Δ*acnAB*, STM Δ*sdhCDAB* and STM Δ*fumABC* were cultured aerobically in LB broth for 18.5 h at 37°C. Cells were harvested and metabolites extracted for subsequent GC-MS analyses. Metabolite levels were normalized to WT levels and means of at least four biological replicates are shown. Background colors indicate the relative concentration of the respective metabolite in the mutant strains compared to WT (red = decreased; green = increased). Gray background indicates indifferent concentrations between STM mutant and WT strains (Student’s *t*-test, *p* < 0.05).

To elucidate the effects of ROS-induced damage of TCA cycle enzymes on the metabolic profile of STM, we deleted multiple Fe-S clusters-containing TCA cycle enzymes and their isoforms without Fe-S clusters, in order to induce a complete block of the catalyzed reaction. Mutant strains defective in both aconitases (Δ*acnA*::FRT Δ*acnB*::FRT, further referred to as STM Δ*acnAB*), or defective in all fumarases (Δ*fumAC*::FRT Δ*fumB*::FRT, further referred to as STM Δ*fumABC*) were generated. Although not conspicuous in proteomic and metabolomic data sets, for completeness all four subunits of succinate dehydrogenase (Δ*sdhCDAB*::FRT, further referred to as STM Δ*sdhCDAB*) were deleted. We performed metabolomics using the same growth conditions as before and compared profiles of TCA cycle mutant strains to WT STM (see Figure 3) regarding glycolysis, TCA cycle and PPP. Glycolysis intermediates were highly reduced for STM Δ*acnAB*, indicating strong similarity to the metabolic profile of STM Δ*sodAB*. Glucose-6-phosphate (G6P) was the most severely reduced metabolite (14.29-fold decreased). STM Δ*sdhCDAB* and Δ*fumABC* showed fewer changes regarding concentrations of glycolysis intermediates. Whereas Δ*sdhCDAB* had reduced amounts of fructose-1,6-bisphosphate (F1,6P_2_; 2.0-fold), glycerate-2-phosphate (G2P; 1.43-fold) and phosphoenolpyruvate (PEP; 2.17-fold), Δ*fumABC* showed only reduced levels of DHAP (2.63-fold). Additionally, both mutant strains showed increased levels of few intermediates: STM Δ*sdhCDAB* accumulated fructose-6-phosphate (F6P; 1.46-fold) and pyruvate (2.84-fold), whereas Δ*fumABC* had even higher accumulations of G6P (2.28-fold) and F6P (1.91-fold).

Concerning the detected TCA cycle intermediates, we again observed similarities between STM Δ*sodAB* and Δ*acnAB*. Total deletion of aconitases resulted in strong accumulation of citrate (99.62-fold) and cis-aconitate (28.02-fold). Although the enzymes catalyzing the reaction from citrate to isocitrate were deleted in STM Δ*acnAB*, isocitrate levels showed the highest increase (170.24-fold compared to WT). A similar increase was observed for STM Δ*sodAB*. Concentrations of α-ketoglutarate (α-KG), succinate and fumarate were more reduced in Δ*acnAB* compared to Δ*sodAB* and we observed a slight accumulation of malate (1.45-fold) for STM Δ*acnAB*. In Δ*sdhCDAB* the abundance of almost all TCA cycle metabolites was reduced despite succinate (1.92-fold increased), due to missing succinate dehydrogenases, catalyzing the reaction from succinate to fumarate, and malate (1.79-fold increased). Interestingly, STM Δ*fumABC* has the fewest changes compared to WT. Besides the high accumulation of fumarate (115.14-fold) due to block of the respective reaction step from fumarate to malate, we observed only a slight reduction of cis-aconitate (1.51-fold).

In addition, concentrations of PPP intermediates were strongly reduced in STM Δ*acnAB* and Δ*sodAB*. Both mutant strains showed same tendencies, with more pronounced reductions in PPP intermediates for STM Δ*acnAB*. In STM Δ*sdhCDAB* only two intermediates were reduced, i.e., ribose-5-P (R5P, 1.28-fold) and sedoheptulose-7-P (S7P, 1.58-fold). Interestingly, only for STM Δ*fumABC* we determined higher levels of the PPP intermediates gluconate (3.93-fold), glucono-1,5-lactone (2.72-fold) and S7P (2.22-fold).

As C-intermediates of all three mentioned pathways generate precursors for amino acid biosynthesis, we detected also severe changes in abundance of amino acids. The amino acid concentrations of STM Δ*sodAB* were comparable to that of STM Δ*acnAB* or STM Δ*sdhCDAB*. Tendencies were the same for STM Δ*sodAB* and Δ*acnAB* with arginine and proline being more abundant, and citrulline/ornithine, isoleucine, lysine and tryptophan being less abundant in both mutant strains. Alterations in amino acid concentrations were more severe for STM Δ*acnAB*, with the exception of proline which was increased 2-fold in STM Δ*sodAB* compared to STM Δ*acnAB*. Strongly reduced amino acid levels in Δ*acnAB* compared to WT are likely caused by severely reduced flux through the TCA cycle due to aconitase deletion. Important precursors for amino acid biosynthesis are therefore less abundant, influencing the amino acid metabolism and abundance of transporter subunits (Supplementary Figure S3 and Supplementary Figure S4). Comparison of STM Δ*sodAB* with Δ*sdhCDAB* demonstrated same tendencies for homoserine, proline and serine, detected in higher amounts, and isoleucine and tryptophan, being less abundant in both strains. Except for tryptophan, increments and decrements were more severe for STM Δ*sodAB*. The amino acid profiles of STM Δ*sodAB* and Δ*fumABC* did not show any similarities and rather appeared disparate.

Summarizing the metabolomics analyses of all mutant strains, deletion of both aconitases led to a metabolic profile surprisingly similar to that of STM Δ*sodAB*.

### Continuous Oxidative Stress Represses *acnA* Expression and Reduces Abundance of AcnA

**FIGURE 4:**
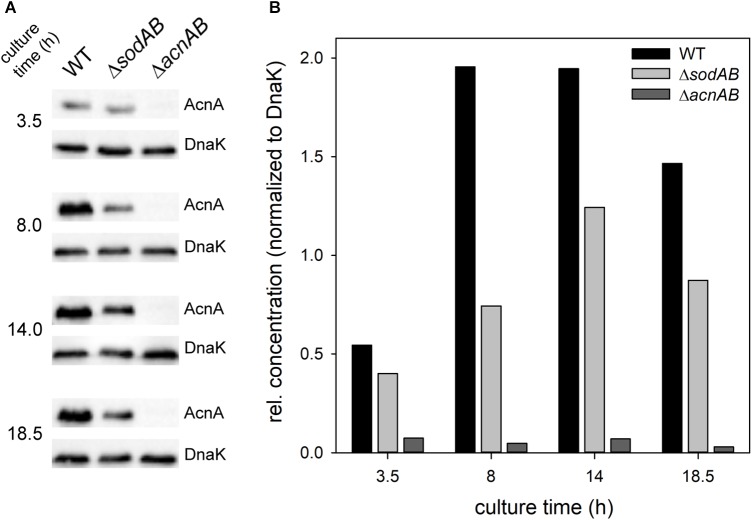
Quantification of relative levels of AcnA in STM WT and Δ*sodAB*. STM WT, Δ*sodAB* and Δ*acnAB* were cultured aerobically in LB broth for 3.5, 8, 14, and 18.5 h at 37°C, cells were harvested and lysed. Proteins were separated using SDS-PAGE on 10% gels and blotted onto nitrocellulose membrane. Blots were incubated with anti-AcnA serum and goat-anti-rabbit-HRP antibody, followed by detection using an ECL kit and Chemidoc system. Blots were stripped and incubated with anti-DnaK antibody and goat-anti-mouse-HRP antibody and subsequent detection. **(A)** Western blot bands of AcnA and DnaK in the respective strains grown for various culture times. **(B)** Quantification of AcnA signals (DnaK normalized) in the respective strains. Depicted values are representative for two biological replicates.

Fe–S cluster containing enzymes such as aconitases are sensitive toward oxidative stress and disassemble upon oxidation of their active center. To evaluate if reduced aconitase levels are a consequence of long cultivation time and therefore continuously rising SOA levels in STM Δ*sodAB*, we conducted Western blot analyses for relative quantification of AcnA amounts over duration of culture (Figure 4). We observed increasing aconitase A amounts in STM WT and Δ*sodAB* from 3.5 to 14 h, and a slight decrease after 18.5 h of growth in LB at 37°C with aeration, however, AcnA levels were lower in the Δ*sodAB* mutant strain at each point of time.

**FIGURE 5:**
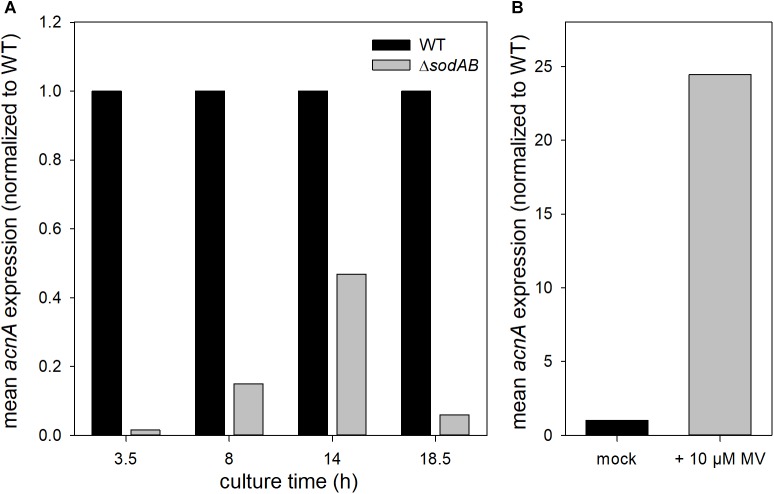
*acnA* expression in STM WT and Δ*sodAB*. STM strains were cultured aerobically in LB broth for **(A)** 3.5, 8, 14, or 18.5 h, or **(B)** 3 h at 37°C, followed by treatment with MV for 30 min. RNA was extracted using hot-phenol method, followed by DNase I digest, cDNA synthesis and qPCR. **(A)** *acnA* expression (16S rRNA normalized) in STM WT and Δ*sodAB* over time (WT = 1). **(B)** *acnA* expression (16S rRNA normalized) in STM WT without (=1) or with MV treatment. Depicted values present results from one of two biological replicates.

Further qPCR experiments revealed that not only AcnA amounts, but also expression of *acnA* is reduced in STM Δ*sodAB* compared to WT at all tested time points (Figure 5A). This result is contradictive to studies which revealed enhanced expression of *acnA* upon short-time exposure to methyl viologen (MV) (Baothman et al., 2013; Seo et al., 2015). STM WT grown for 3.5 h in LB was treated with 10 mM MV for 30 min (Figure 5B). Indeed, we observed a strong increase in *acnA* expression by MV treatment in STM. These results reveal opposite effects of short-term and continuous oxidative stress on *acnA* expression in STM. Reduced AcnA protein levels in STM Δ*sodAB* compared to WT determined by proteomic analyses are likely due to reduced expression. However, metabolic perturbations observed in STM Δ*sodAB* may additionally be explained by ROS-induced damage to sensitive Fe–S clusters of AcnA.

### Metabolic Perturbations of TCA Cycle Enzymes Did Not Affect Sensitivity Toward ROS of STM in Macrophages

A STM strain defective in SOD is known to be sensitive toward oxidative stress and exhibits a reduced virulence in macrophages (Tsolis et al., 1995). Furthermore, STM with deleted aconitases was reported to be sensitive toward oxidative stress (Baothman et al., 2013). Motivated by the strong similarities between the metabolic profiles, we set out to elucidate if metabolic perturbations of the TCA cycle affect tolerance of STM toward oxidative burst of phagocytes. We determined intracellular survival and proliferation in murine macrophages-like RAW264.7 cells, which were activated by interferon-γ to stimulate antimicrobial activities such as oxidative burst. As negative control, we used a strain defective in SsaV, a main component of the *Salmonella* pathogenicity island 2-encoded type III secretion system (SPI2-T3SS), which is unable to translocate effector proteins into the host cell cytosol (Hensel et al., 1997). Several strains with defects in TCA cycle enzymes have been previously analyzed for intracellular phenotypes in macrophages (Tchawa Yimga et al., 2006; Bowden et al., 2010; Wynosky-Dolfi et al., 2014), but analyses of strains defective in both aconitases or all three fumarases are pending.

**FIGURE 6:**
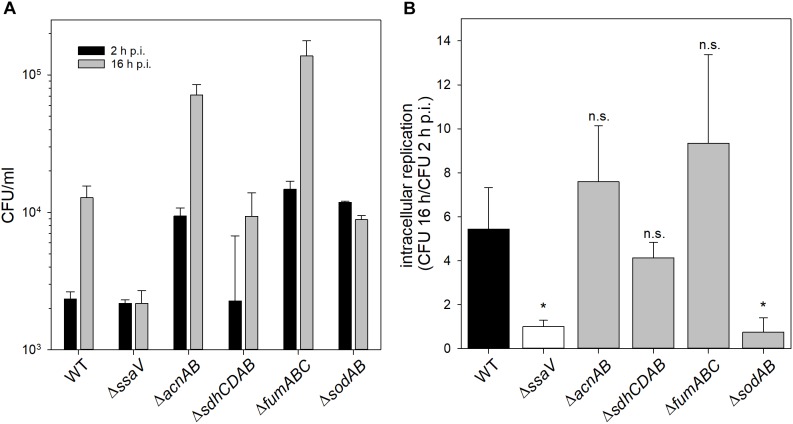
Phagocytosis and intracellular replication of STM *ΔsodAB* and mutant strains with defects in Fe-S cluster-containing TCA cycle enzymes. RAW264.7 macrophages were activated with 5 ng/ml interferon-γ 24 h prior infection. STM strains were grown aerobically o/n in LB broth at 37°C and used for infection at a MOI of 1. Infection was synchronized by centrifugation for 5 min. After infection for 25 min, non-internalized bacteria were removed by washing and remaining extracellular bacteria were killed by gentamicin treatment (1 h at 100 μg/ml, followed by 10 μg/ml for the remaining time). Host cells were lysed 2 and 16 h p.i. with 0.1% Triton X-100 in PBS and lysates were plated onto MH agar plates to determine the CFU of intracellular STM. **(A)** Depicted are CFU/ml obtained at 2 and 16 h p.i. **(B)** X-fold replication was determined as ratio of CFU at 2 and 16 h p.i. One experiment representative for three biological replicates is shown. Statistical analyses were performed by Student’s *t*-test and significances are indicated as follows: ^∗^*p* < 0.05; n.s., not significant.

We determined strongly increased phagocytosis for Δ*sodAB*, Δ*acnAB* and STM Δ*fumABC* compared to WT and STM Δ*ssaV* (see Supplementary Figure S5). Intracellular replication of STM Δ*sodAB* at 16 h p.i. was even lower than at 2 h p.i., similar to highly attenuated STM Δ*ssaV* (see Figure 6). Although Δ*sodAB* and STM Δ*acnAB* have rather similar metabolic profiles and increased HPO sensitivity, STM Δ*acnAB* was not reduced in intracellular replication. These results indicate that STM Δ*sodAB* is unable to proliferate and shows reduced survival in the harsh intracellular environment of activated macrophages, whereas the TCA cycle mutant strains were not impaired. Similar results were obtained from survival assays of STM WT, Δ*ssaV*, Δ*acnAB*, Δ*sdhCDAB*, Δ*fumABC*, and Δ*sodAB* after methyl viologen treatment *in vitro* (see Supplementary Figure S6). Thus, metabolic perturbations of the TCA cycle do not influence the sensitivity of STM against ROS in our experimental setup.

### Comparison of the Proteomic Profiles of STM Δ*sodAB* and Δ*acnAB*

**FIGURE 7:**
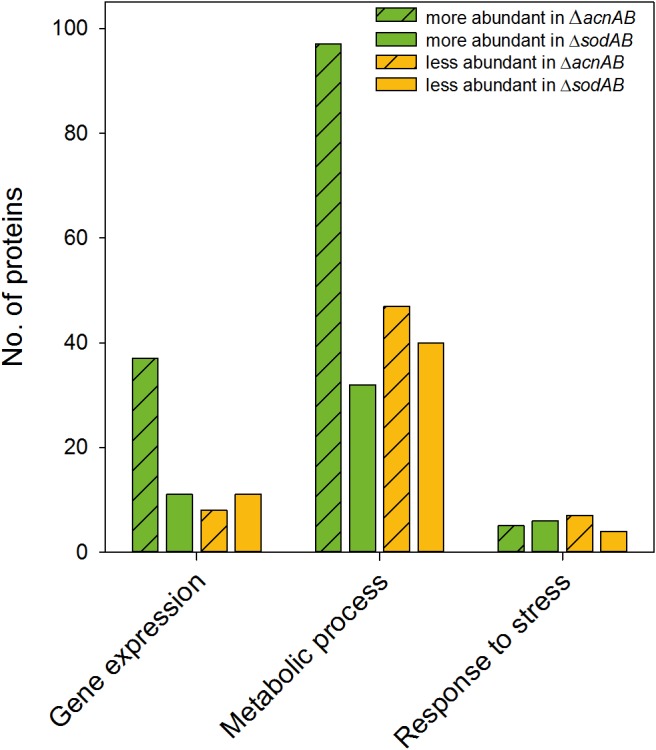
Differentially abundant proteins in Δ*acnAB*- or Δ*sodAB*-deficient STM compared to WT. Detected proteins were classified according to Gene ontology (biological process) ‘gene expression,’ ‘metabolic process’ and ‘response to stress.’ Depicted are numbers of proteins with significantly altered levels in mutant strains compared to WT. Statistical analyses were performed with Student’s *t*-test, *p* < 0.05.

Metabolism and virulence are interconnected in various pathogens. Although STM Δ*acnAB* and Δ*sodAB* exhibited very similar metabolic profiles, the sensitivity toward oxidative burst of activated macrophages and MV differed between both strains. Thus, we analyzed the proteome of STM Δ*acnAB* in order to determine factors leading to the distinct phenotype (see Supplementary Table S3). Main factors influencing replication are gene expression in response to environmental cues, metabolic activity, and the ability to cope with stress. Therefore, we categorized all differentially abundant proteins of STM Δ*acnAB* and Δ*sodAB* compared to WT according to gene ontologies (biological process) ‘gene expression,’ ‘metabolic process’ and ‘response to stress’ (Figure 7). Considering proteins related to gene expression, we determined 37 proteins being more abundant in Δ*acnAB* compared to WT, whereas only eight proteins were reduced. In contrast, Δ*sodAB* shows 11 proteins being more abundant and the same number being less abundant, indicating that STM Δ*acnAB* is less impaired in replication *in vitro*, and thus likely less attenuated *in vivo*. The abundance of metabolic proteins is very different in both strains. Under *in vitro* conditions, in STM Δ*acnAB* 97 proteins were more abundant compared to WT and only 32 for STM Δ*sodAB*. Furthermore, for the *sodAB*-deficient strain we detected more metabolic proteins in reduced amounts. Especially the abundance of enzymes involved in CCM were increased in STM Δ*acnAB* (Supplementary Figure S7). Focusing on proteins related to stress response, we expected to detect severe differences between both mutant strains. However, we found that a comparable number of proteins were differentially expressed in both mutants strains compared to WT. As this result was unexpected, we analyzed this group in more detail for STM Δ*acnAB* (see Table 3). Five proteins were more abundant in Δ*acnAB* compared to WT: Clp (chaperone protein: 1.17-fold), TrxB (thioredoxin reductase: 1.31-fold), AhpC (alkyl hydroperoxide reductase C: 1.54-fold), RecA (protein RecA: 1.80-fold), and SodA (superoxide dismutase A: 1.97-fold). Six proteins were decreased in their abundance in 1.26- to 2.43-fold levels. For STM Δ*sodAB* we detected much higher increments in the range of 3.11- to 12.56-fold. Thus, due to the endogenous accumulation of SOA, STM Δ*sodAB* seems to be exposed to much higher stress levels than STM Δ*acnAB*. Under intracellular conditions, stress levels will be even higher due to antimicrobial activities of the host cell. We conclude that reduced intracellular replication of Δ*sodAB* is due to inability to cope with oxidative stress in a sufficient manner, but not due to ROS-mediated metabolic perturbations.

**Table 3:** Differentially abundant stress-response proteins of STM Δ*acnAB* in comparison to WT.^∗^

	Gene product	Protein description	Ratio Δ*acnAB*/WT
Less abundant	YciF	Putative cytoplasmic protein	0.41
in STM	OsmC	Putative resistance protein	0.50
Δ*acnAB*	TrxA	Thioredoxin	0.50
	HtrA	Periplasmic serine endoprotease	0.59
	Dps	DNA protection during starvation protein	0.60
	DnaJ	Chaperone protein	0.63
	SodB	Superoxide dismutase	0.79
More abundant	ClpB	Chaperone protein	1.17
in STM	TrxB	Thioredoxin reductase	1.31
Δ*acnAB*	AhpC	Alkyl hydroperoxide reductase C	1.54
	RecA	Protein RecA	1.80
	SodA	Superoxide dismutase	1.97

*^∗^Listed are proteins classified in category ‘stress response’ according to Gene Ontology (biological process). Only proteins with significantly different amounts are depicted (Student’s t-test, p < 0.05).*

## Discussion

Reactive oxygen species (ROS) are generated intrinsically in bacteria (i) by the respiratory chain, (ii) by indirect action of antibiotics, or (iii) are produced by phagocytes combating pathogens. ROS damage DNA and other macromolecules, resulting in growth restriction and/or killing of bacteria. We elucidate how oxidative stress-induced metabolic perturbations of the primary metabolism reduce bacterial viability and facilitate clearance of pathogens. For the analysis of long-term exposition of STM to ROS we used a mutant strain defective in both cytosolic SODs. Since all three types of ROS should accumulate in STM Δ*sodAB*, we expected to measure increased abundance of proteins encoded by members of the SoxRS- and OxyRS-regulon, induced by SOA and HPO, respectively (Seo et al., 2015). We demonstrated that long-term oxidative stress in a *sodAB* deletion strain induces metabolic perturbations in amino acid metabolism and CCM. Metabolic profiling proved the high influence of aconitase inhibition and reduced *acnA* expression on STM metabolism under oxidative stress, in addition to amino acid auxotrophies reported earlier. As deletion of aconitases, fumarases or succinate dehydrogenases alone did not influence *Salmonella* virulence in RAW264.7 macrophages or survival of MV (=paraquat) treatment, we conclude that inactivation of those metabolic enzymes does not reduce bacterial viability under oxidative stress. However, although the metabolic profile of Δ*sodAB* appeared mainly influenced by inactivated TCA cycle enzymes, cumulative effects of different metabolic defects induced by oxidative stress might have a higher impact.

Significant higher amounts were detected for six proteins in STM Δ*sodAB* compared to WT that are involved in oxidative stress response and DNA damage repair but are regulated by other factors than SoxRS and OxyRS. Two members of the OxyRS regulon, SufS and GrxA, were detected for STM Δ*sodAB*, while AhpC and Dps were determined in higher levels in STM Δ*sodAB*, but not in a significant manner. However, in studies analyzing effects of oxidative stress on *E. coli* and STM, bacteria were treated with very high concentration of HPO or MV. Thus, other regulation mechanisms might dominate if oxidative stress is continuous. Furthermore, Gu and Imlay (2011) observed that paraquat directly oxidizes SoxR, and that SOA is a weak inducer of the SoxRS regulon. Our data demonstrate that SOA does not increase levels of SoxRS-regulated proteins, at least if long-term exposition occurred. This is in line with our observation of reduced *acnA* expression in STM Δ*sodAB*. Whereas short-term oxidative stress increases *acnA* expression in term of SoxRS-regulation, long-term oxidative stress leads to contrary effects. Studies on oxidative stress response of EHEC *E. coli* O157:H7 demonstrated that stationary phase bacteria were induced poorly when exposed to paraquat. Allen and Griffiths (2012) postulated that stationary phase bacteria are more resistant toward oxidative stress, as general stress-related proteins are already increased in a RpoS-dependent manner. As we analyzed long-term oxidative stress, our data are in line with these results.

Weak induction of SoxRS is one explanation for the differences our metabolic analyses revealed in comparison to studies published on short-term stressed *E. coli*. Besides *acnA* expression, expression of glucose-6-phosphate-1-dehydrogenase (Zwf), important for the redirection of the metabolic flux toward PPP, can be accelerated by SoxRS (Seo et al., 2015). By increased flux through PPP, the concentration of NADPH rises, which ensures proper regeneration of thioredoxins and glutaredoxins, required for neutralization of ROS (Seo et al., 2015; Christodoulou et al., 2018). However, although STM Δ*sodAB* did not increase metabolic flux toward PPP, concentrations of glycolysis intermediates were also low. Thus, we observed a globally reduced metabolic activity of long-term ROS-stressed STM.

We aimed to elucidate which significance ROS-mediated defects of metabolic enzymes have on the deleterious effects of oxidative stress in STM. The damage of Fe-S clusters upon ROS exposure are well known, and are reflected by our proteomic data (reduced level of AcnA and FumA for STM Δ*sodAB* compared to WT). Data obtained for STM Δ*sodAB* clearly demonstrate that long-term oxidative stress leads to enhanced abundance of TCA cycle enzymes, i.e., isocitrate dehydrogenase (IcdA), succinate-CoA-ligase (SucD) and malate dehydrogenase (Mdh). Again, we did not detect an increased abundance of SoxRS regulon members AcnA and FumC (Liochev and Fridovich, 1992; Gruer and Guest, 1994).

The similarities determined between metabolic profiles of STM Δ*sodAB* and Δ*acnAB* are striking. Both mutant strains showed decreased metabolite levels of glycolysis, PPP and TCA cycle, despite of citrate, cis-aconitate and isocitrate. Thus, although the range of metabolic enzymes being attacked by ROS is wide, decreased aconitase abundance and likely activity acts as the main factor influencing metabolic perturbations in ROS-stressed STM in our experimental setup.

Whereas isocitrate accumulation in STM Δ*sodAB* is most likely induced by inhibition of isocitrate dehydrogenase during exposure to ROS (Lee et al., 2001; Rui et al., 2010), this phenotype is quite counterintuitive for an aconitase-deficient strain, incapable to catalyze the formation of isocitrate from citrate. The reaction to isocitrate is of endergonic nature, but due to high accumulation of citrate, we assume spontaneous conversions. Nonetheless, like in STM Δ*sodAB*, isocitrate accumulation in STM Δ*acnAB* requires inhibition of the proceeding metabolic step to α-KG. Phosphorylation of ICDH, and in consequence inhibition of the enzyme is decreased by isocitrate (Nimmo and Nimmo, 1984), thus this mechanism is not considered to take place in STM Δ*acnAB*.

The surprisingly broad impact of aconitase inactivation on the overall metabolism of ROS-stressed cells led us to comparison of proteomic profiles of Δ*acnAB* and Δ*sodAB* strains. Although the metabolic profiles showed same tendencies, abundances of CCM proteins were different. Surprisingly, all three pathways showed increased levels of enzymes in STM Δ*acnAB* strain compared to WT. Furthermore, we determined Zwf in Δ*acnAB*, but not in WT, indicating elevated levels. As *zwf* expression can be augmented by SoxRS (Lundberg et al., 1999), we re-considered stress response proteins of STM Δ*acnAB* compared to WT. For *E. coli* it was reported that Δ*acnAB* strains show increased levels of SodA and other stress proteins like TrxB. These observations were explained by the dual function of aconitases (Tang et al., 2002). ROS inactivated aconitases function as apo-enzymes and influence mRNA stability and translation. The authors proved an inhibitory effect of apo-AcnB and a stabilizing effect of apo-AcnA on *sodA* mRNA. However, in *E. coli* Δ*acnAB*, expression of the OxyRS regulon was not strongly increased (Tang et al., 2002). We determined a significantly enhanced abundance of AhpC in STM Δ*acnAB*. Additionally, KatG and AhpF were detected in STM Δ*acnAB*, but not in WT, thus we hypothesize increased expression of OxyRS-regulated genes. As HPO is the inducer of the OxyRS regulon (Zheng et al., 1998), the increased level of SodA determined for STM Δ*acnAB*, might enhance HPO levels and influence regulon induction. Another possibility is the antimicrobial effect of citrate. Citrate is used as food preservative and growth-inhibiting activity was proven against Gram-positive and Gram-negative bacteria (Nagaoka et al., 2010). The precise mechanism has not been unraveled, but citrate acts as chelator similar to EDTA. Based on the observation that citrate-treated *Streptococcus pneumoniae* cells showed lysis and abnormal cell morphologies, the main hypothesis is that citrate chelates cations, leading to disruption of the cytoplasmic membrane (Nagaoka et al., 2010).

The effects of citrate were determined in more detail for mammalian cells. In eukaryotes citrate is known to inhibit phosphofructokinase 1 and 6-phosphofructo-2-kinase/fructose-2,6-bisphosphatases. Furthermore, by decreasing fructose-1,6-bisphosphate levels, citrate may indirectly inhibit pyruvate kinase. With respect to the TCA cycle, citrate was reported to inhibit pyruvate dehydrogenase and succinate dehydrogenase, leading to enhanced gluconeogenesis (reviewed in Iacobazzi and Infantino, 2014). Furthermore, citrate is reported to increase oxidative stress in the presence of HPO and likely SOA, by raising hydroxyl radical formation (Gutteridge, 1990). This was explained by increased release of iron from ferritin, as this process depends on chelators like citrate (van de Wier et al., 2013).

Transferring these results to our bacterial model, an inhibitory effect of citrate on glycolytic and TCA cycle enzymes is possible and could explain reduced metabolite levels despite increased enzyme abundances in STM Δ*acnAB*. Furthermore, accumulated citrate would enhance ROS concentrations and OxyRS-regulated gene expression as observed for STM Δ*acnAB* and Δ*sodAB*.

**FIGURE 8:**
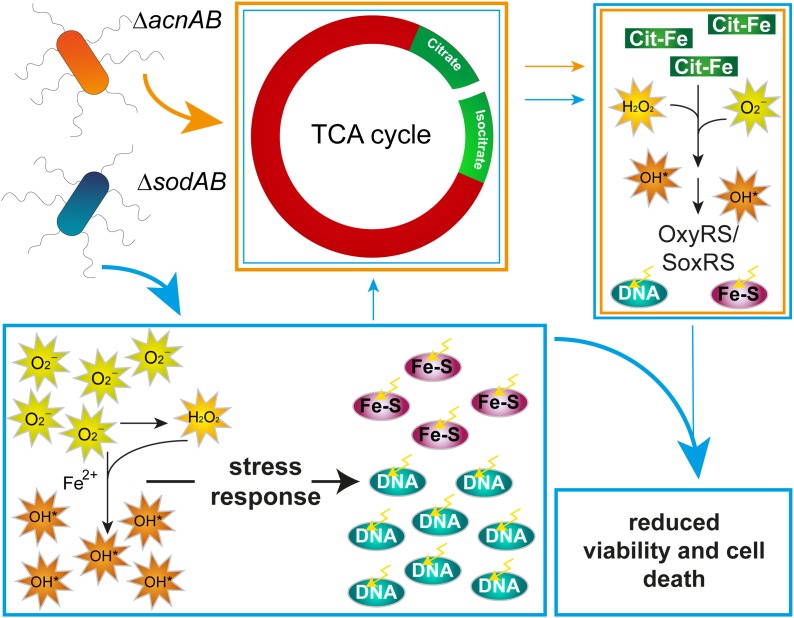
Model summarizing effects of aconitase or superoxide dismutase deletion on STM physiology. Deletion of aconitases disrupts the TCA cycle, leading to citrate and, by an unknown mechanism, isocitrate accumulation. Citrate acts as chelator of iron, among other ions, which reacts in Fenton and Haber-Weiss reactions to HR. Increased ROS concentration induce stress regulons, minimizing effects of ROS damage. Deletion of cytosolic superoxide dismutases leads to ROS stress by accumulating SOA. SOA dismutate in part to HPO. Both kinds of ROS damage Fe–S clusters and form with liberated iron ions HR. Although stress response proteins are induced, continuous oxidative stress overwhelms the capacity of ROS-detoxifying enzymes. Beside reduced expression of *acnA*, ROS attack Fe–S clusters, including those of aconitases, mediating citrate accumulation and downstream effects similar to those observed in STM Δ*acnAB*. In STM Δ*sodAB* ROS from both sources damage macromolecules like DNA, which reduces bacterial viability.

To summarize these aspects, long-term exposure of STM to oxidative stress results in a similar metabolic profile as observed for STM Δ*acnAB*. As ROS damage Fe-S clusters, AcnA expression, abundance and possibly activity is reduced in Δ*sodAB*, leading to citrate accumulation. Further similarities between both metabolic profiles can be reasoned, (i) by effects of reduced *acnA* expression and AcnA inhibition and in consequence citrate accumulation in STM Δ*sodAB*, or (ii) by ROS stress, which also arises in Δ*acnAB* due to hydroxyl radical formation by extreme citrate accumulation. However, these metabolic defects are not the crucial point for reduced survival of STM Δ*sodAB* upon additional ROS attacks by macrophages or MV treatment. Therefore, DNA damage and attacks on various macromolecules are the main factors influencing bacterial viability (Figure 8). The increased phagocytic uptake of both mutant strains and additionally STM Δ*fumABC* was a striking observation and has to be elucidated further. Our experimental setup indicates that ROS-induced metabolic defects can accelerate oxidative stress STM is exposed to. Nutrient-restricted environments and especially utilization of glycerol as main carbon source in the mouse model (Steeb et al., 2013) could enhance the effects observed in our studies.

## Materials and Methods

### Bacterial Strains

*Salmonella enterica* serovar Typhimurium NCTC 12023 was used as wild-type (WT) strain. Isogenic mutant strains were constructed by aaa Red-mediated mutagenesis (Datsenko and Wanner, 2000) using pKD13 as template plasmid. Strains used in this study are listed in Table 4. Primers and plasmids required for mutagenesis, removal of resistance cassettes, and check PCR for correct insertion are listed in Supplementary Table S4. Mutant alleles were transferred into fresh strain background or combined with other mutations via P22 transduction as described before (Popp et al., 2015).

**Table 4:** Bacterial strains used in this study.

Designation	Genotype	Relevant defect(s)	References
NCTC 12023	Wild-type	–	NCTC, Colindale, United Kingdom
MvP1564	Δ*fumAC*::FRT Δ*fumB*::FRT	Fumarase A, B and C	This study
MvP2400	Δ*sodA*::FRT Δ*sodB*::FRT	Superoxide dismutase A and B	This study
MvP1576	Δ*acnA*::FRT Δ*acnB*::FRT	Aconitase A and B	This study
MvP1523	Δ*sdhCDAB*::FRT	Succinate dehydrogenase subunit A, B, C, and D	This study
MvP1890	Δ*ssaV*::FRT	Component of the SPI2-T3SS	This study
MvP2785	Δ*ilvA*::*aph*	Threonine dehydratase	This study
MvP2786	Δ*trpC*::*aph*	Tryptophan biosynthesis protein	This study

### Growth Conditions

Bacteria were grown as described (Popp et al., 2015). For growth curves, LB broth or PCN medium (pH 7.4) containing 80 mM MOPS (3-morpholinopropane-1-sulfonic acid) and 25 mM phosphate or (pH 5.8) containing 80 mM MES [2-(N-morpholino)ethanesulfonic acid] and 0.4 mM phosphate were used. For amino acid supplementation, valine (0.6 mM), leucine (0.8 mM), isoleucine (0.4 mM), methionine (0.2 mM), cysteine (0.1 mM), histidine (0.2 mM), phenylalanine (0.4 mM), tryptophan (0.1 mM), and tyrosine (0.2 mM) were added to PCN medium. For growth analyses of STM Δ*acnAB*, succinate (25 mM) and glutamine (4 mM) were supplemented to the medium.

### Growth Kinetics

*Salmonella enterica* serovar Typhimurium strains were cultured o/n in LB broth at 37°C with aeration. For growth curves in LB broth, OD_600_ was determined and 25 ml fresh broth was inoculated to an OD_600_ of 0.01. For growth curves in minimal PCN broth, cells of 1 ml LB o/n cultures were pelleted by centrifugation (7,000 × g, 5 min). The supernatant was discarded, the pellet washed by resuspension in PBS and one further centrifugation step. Bacteria were resuspended in 1 ml PCN medium with or without amino acids or succinate and glutamine supplementation. OD_600_ was determined and 25 ml fresh broth, with and without supplementation was inoculated to an OD_600_ of 0.01. Optical density was measured in hourly intervals from 0 to 8 h.

### Growth Tests With Auxotrophic Mutant Strains

*Salmonella enterica* serovar Typhimurium WT, Δ*sodAB*, Δ*ilvA*, and Δ*trpC* were grown o/n in LB broth at 37°C with aeration. Bacteria were pelleted by centrifugation (20,000 × g, 5 min), and spent supernatants were filter sterilized. Aliquots of 3 ml of each supernatant were inoculated with LB o/n cultures of the four respective strains to an OD_600_ of 0.01 and incubated again at 37°C with aeration. After 24 h of culture, OD_600_ was determined.

### Culture Conditions and Cell Harvest for Metabolite Profiling by GC-MS

For each strain, o/n cultures in LB broth were used to inoculate 25 ml LB broth to an OD_600_ of 0.01 and cultured for 18.5 h at 37°C with agitation at 180 rpm. For measurements of metabolites in bacterial cells, 5 ml culture were transferred onto Durapore PVDF filter membranes (Merck, Darmstadt, Germany) with a pore size of 0.45 μm by suction. After washing with PBS, cells were scraped from the filter into 1 ml of fresh PBS. Bacteria were pelleted (1 min, at 22,000 × g) and the supernatant discarded, before shock-frozen in liquid nitrogen. Afterward samples were freeze-dried and their dry weights were determined.

### GC-MS Sample Preparation and Measurement

Metabolome analysis of the TCA cycle mutant strains was performed by GC-MS using protocols according to Plassmeier et al. (2007). For metabolite extraction 1 ml 80% methanol containing 10 μM ribitol (RI, internal standard) and 500 mg acid-washed glass beads (Sigma-Aldrich, United States) were added to the dried samples. Disruption occurred in three cycle intervals for 1 min with 6,200 rpm using a homogenizer (Precellys, Peqlab). Samples were centrifuged at 18,500 × g for 20 min, supernatants were evaporated in a nitrogen stream. For derivatization, 50 μl of 20 mg/ml of methoxylamine hydrochloride in pyridine were added to each sample and incubated with constant stirring at 37°C for 90 min. Next 50 μl of N-methyl-N-[trimethylsilyl]-trifluoroacetamide were added and incubated at 37°C for further 30 min. After addition of 10 μl RI standard and further 5 min incubation at 37°C, samples were centrifuged at 3,220 × g at RT for 5 min. Subsequently, supernatants were used for GC-MS measurement using a TraceGC gas chromatograph equipped with a PolarisQ ion trap and an AS1000 autosampler (Thermo Finnigan, Dreieich, Germany) according to Plassmeier et al. (2007). Metabolite quantities were normalized to ribitol and dry weights of used samples as described in Plassmeier et al. (2007). Mean relative pool size changes of the mutant strains compared to WT were calculated and only those data with an error probability (Student’s *t*-test) of less than 0.05 were used for further interpretation.

### Proteome Profiling via LC-MS Measurement

Bacteria were cultured as described for metabolite profiling. Samples were kept on ice during the protein isolation and all centrifugation steps occurred at 4°C, if not stated otherwise. For proteomic comparison of STM Δ*acnAB* versus WT, cells from 50 ml o/n culture were pelleted by centrifugation (9,000 × g, 10 min). The pellet was washed twice with 25 ml pre-cooled PBS before resuspension in lysis buffer [50 mM Tris, pH 8.5, 1% SDS, protease inhibitor (cOmpleteTM ULTRA tablets, mini, EDTA-free, Sigma-Aldrich)] supplemented with 500 mg zirconia/silica beads (0.1 mm diameter, biospec products) per sample. Cell disruption occurred in five cycles for 1 min with maximal speed, using a Vortex Genie 2 equipped with a microtube holder (Scientific Industries). Cell debris were removed by centrifugation (14,000 × g, 10 min at 15°C) and proteins from the supernatant were precipitated with 10% TCA at 4°C o/n. Afterward proteins were pelleted by centrifugation (14,000 × g, 10 min) and the resulting protein pellet was washed with 100 μl 70% acetone at 4°C for 30 min before air drying.

For proteomic analysis of STM Δ*sodAB* and WT, about 6 × 10^9^ bacteria were pelleted by centrifugation (9,000 × *g*, 10 min). Supernatant was removed and the bacterial pellet frozen in liquid nitrogen. Protein was isolated using Trizol (Thermo Fisher Scientific), according the manufacturers’ instruction. Air-dried protein pellets from either extractions were resuspended in 50 mM ammonium bicarbonate buffer (pH 8.0) containing 1% SDS, before reduced and alkylated with 5 mM DTT and 20 mM iodoacetamide, respectively. Subsequently the samples were rebuffered into 50 mM ammonium bicarbonate buffer on filter columns (molecular weight cut off 10 kDa, Amicon, Millipore). Protein amounts of all samples were determined by Pierce BCA protein assay kit. Protein digest and LC-MS measurement was performed as described in Hansmeier et al. (2018). Briefly, proteins were digested using Trypsin Gold (Promega, Madison**, WI, United States**) according manufacturers**’** instruction before vacuum drying. Dried digested samples were resuspended in 0.1% formic acid and 3% acetonitrile. For calibration STM WT and Δ*sodAB* samples were spiked with rabbit phosphorylase (Waters Corporation, Milford, MA**, United States**), whereas the other samples were internally calibrated using Ef-Tu as reference. Each sample was analyzed via a Waters NanoAcquity system coupled to a Waters Synapt G2 HDMS. A Waters NanoAcquity UPLC Symmetry C18 trap column (180 μm × 20 mm, dp: 5 μm) was used for desalting and focusing of peptides prior to their elution onto the Waters Acquity UPLC M-class HSS T3 analytical column (75 μm × 200 mm, dp: 1.8 μm) using a 120 min gradient from 3% acetonitrile/0.1% formic acid to 45% acetonitrile/0.1% formic acid at a flow rate of 0.35 μl/min Eluting peptides were analyzed in positive MS^E^ resolution mode with 1 s scan time. Collision energy was set as before. To ensure mass accuracy, leucine enkephaline was measured as lock mass every 30 s. The resulting spectra were processed with the ProteinLynx Global Server (PLGS) v. 3.02 (WT vs. Δ*sodAB*) or v. 2.2.5 (WT vs. Δ*acnAB*) with Identity (Waters) and searched against a protein sequence database build with the Uniprot *Salmonella* reference proteome (downloaded March 2015), supplemented with the sequences of the Waters PhosB standard for quantitation. Search parameter were set to mass tolerance (8 ppm), trypsin specificity, 1 missed cleavage, stable modification carbamidomethyl, variable modification methionine oxidation, false discovery rate 1%. To adjust for variances between injections, the concentration values for each chromatographic run were normalized against the total femtomole of protein quanti**fi**ed per analysis. To determine signi**fi**cantly di**ff**erential protein amounts in STM mutants compared to WT, we deployed Student’s *t*-test and used the Benjamini-Hochberg method to adjust for multiple hypothesis testing.

### Gentamicin Protection Assay

Culture and infection of RAW264.7 macrophage cell line was performed as described (Popp et al., 2015). Briefly, RAW264.7 macrophages were infected with STM o/n cultures with a MOI of 1, centrifuged 5 min at 370 × g to synchronize infection, and infection proceeded for 25 min. Cells were washed thrice with PBS and extracellular, non-phagocytosed bacteria were killed by incubation with medium containing gentamicin (100 μg/ml for 1 h, followed by 10 μg/ml for the remaining incubation). At 2 and 16 h p.i., cells were washed thrice with PBS and lysed by addition of 0.1% Triton X-100 in PBS. Serial dilutions of the inoculum and lysates were plated on Mueller-Hinton II agar plates, incubated o/n at 37°C, and CFU were determined. The phagocytosis rate was determined as percentage of internalized bacteria of the inoculum. The x-fold replication rates were calculated as quotient of the CFU/ml obtained at 16 and 2 h p.i.

### Methyl Viologen Survival Assay

*Salmonella enterica* serovar Typhimurium strains were cultured o/n in LB broth at 37°C and 6 × 10^5^ bacteria/ml in PBS were exposed to methyl viologen to a final concentration of 10 mM for 2 h at RT without shaking. The number of viable bacteria/ml before (=inoculum) and after methyl viologen treatment were determined by plating serial dilutions on MH agar plates. Survival rates were calculated as percentage of viable bacteria as fraction of the inoculum.

### Western Blot Analysis

*Salmonella enterica* serovar Typhimurium strains were grown o/n in LB at 37°C with aeration. OD_600_ was determined and 25 ml fresh LB broth was inoculated to an OD_600_ of 0.01. Cultures were incubated in a shaking water bath at 37°C and 180 rpm. After 3.5, 8, 14, and 18.5 h of growth, 400 μl of culture were collected and cells harvested by centrifugation for 10 min at 20,000 × g. Pelleted bacteria were resuspended in SDS loading buffer and incubated for 10 min at 100°C. Ten μl were used for SDS PAGE on 10% gels. Proteins were blotted onto a 0.45 μm nitrocellulose membrane according to the Kyle-Anderson semi dry procedure. Blots were incubated with antiserum against AcnA (1:1,000 dilution, kindly provided by Prof. Dr. Jeff Green) and detected by further incubation with goat anti rabbit IgG antibody conjugated to HRP (1:5,000 dilution). Blots were developed using an ECL detection kit (Thermo Fisher). Visualization was conducted with a Chemidoc imaging system (Bio-Rad). For normalization of detected signals, blots were stripped with 5% TCA and incubated with antibody against DnaK (1:5,000 dilution), followed by incubation with goat anti mouse IgG antibody conjugated to HRP (1:10,000 dilution) and visualization. Quantification of signals was conducted using ImageLab software (Biorad).

### qPCR

*Salmonella enterica* serovar Typhimurium strains were cultured as described for Western blot analyses. After 3.5, 8, 14, and 18.5 h of growth, 1.2 × 10^9^ bacteria were treated with stop-solution (95% EtOH, 5% phenol saturated with 0.1 M citrate buffer, pH 4.3, Sigma-Aldrich) and snap-frozen in liquid nitrogen. Samples were thawed on ice, centrifuged (13,000 × g, 4°C, 10 min), supernatant discarded and the pellets stored at -70°C. As control for inducible *acnA* expression, STM WT was grown for 3 h in LB broth at 37°C with aeration. 1.2 × 10^9^ bacteria were pelleted and resuspended in PBS with or without 10 mM methyl viologen. Cell suspensions were incubated for further 30 min at 37°C followed by treatment with stop-solution as described before.

RNA was prepared according to the ‘hot phenol’ method (Mattatall and Sanderson, 1996; Sittka et al., 2009). In brief, cells were lysed using a lysis buffer (0.5 mg/ml lysozyme (Sigma-Aldrich) in TE buffer pH 8.0 (Promega) with 2% SDS (Sigma-Aldrich). Afterward, samples were buffered with 3 M sodium acetate buffer (pH 5.2) (Life technologies), before RNA was extracted with Roti-Aqua phenol (Roth) at 64°C. After centrifugation (15.000 × g, 4°C, 20 min), the watery phase was loaded on heavy phase lock gel tubes (5PRIME, Hilden) supplemented with chloroform for further purification. Nucleic acids were precipitated twice with a 30:1 mixture of absolute ethanol and 3 M NaOAc (pH 5.2) with o/n incubation steps at -20°C. Pellets were washed with 75% ethanol, air-dried and solved in RNase-free water. Samples were treated with RNase-free DNase I (NEB), before RNA concentrations were determined using a nano-photometer (Implen). cDNA synthesis was performed with the RevertAid First strand cDNA synthesis kit (Thermo Fisher), using 1 μg RNA and random hexamer primers. qPCR was performed with the Maxima SYBR Green/Fluorescein qPCR Master Mix (Thermo Fisher) using the iCycler with MyiQ module (Bio-Rad). Data were normalized (16S rRNA) calculated in consideration of primer efficiencies determined using serial dilutions of cDNA. Oligonucleotides used in this study are listed in Supplementary Table S4.

## Author Contributions

JN, MH, and NH designed the research. JN, MP, T-CC, LK, BH, and NH performed the research. JN, MP, T-CC, LK, BH, MH, and NH analyzed the data. JN, T-CC, MH, and NH wrote the manuscript with input from all authors.

## Conflict of Interest Statement

The authors declare that the research was conducted in the absence of any commercial or financial relationships that could be construed as a potential conflict of interest.

## Supplementary Material

FIGURE S1 Growth kinetics of STM WT, Δ*sodAB* and Δ*acnAB* in rich and minimal media. STM strains were pre-cultured o/n in LB broth at 37°C with aeration. (A) OD_600_ was determined and 25 ml fresh broth was inoculated to an OD_600_ of 0.01. (B,C) cells of 1 ml LB o/n cultures were pelleted by centrifugation. The supernatant was discarded, the pellet washed by resuspension in PBS and one further centrifugation step. Bacteria were resuspended in 1 ml PCN medium (B) pH 5.8 and 0.4 mM phosphate or (C) pH 7.4 and 25 mM phosphate with or without supplementation with amino acids (=aa; see Material and Methods), glutamine (=glu; 4 mM) or succinate (=succ; 25 mM). OD_600_ was determined and 25 ml fresh broth, with and without supplementation, was inoculated to an OD_600_ of 0.01. Optical density was measured in hourly intervals from 0 to 8 h. Data present average values of two biological replicates with the respective standard deviation.

FIGURE S2 Growth of STM WT, Δ*sodAB* and Δ*acnAB* under anaerobic conditions. STM strains were cultured o/n in LB broth at 37°C with aeration. Pelleting, washing steps and inoculation was performed as described for Supplementary Figure S1, but using 3 ml culture in 15 ml conical tubes. Cultures were covered with 1 ml paraffin oil, and lids were closed and wrapped with parafilm. Cultures were grown statically at 37°C. Optical density was measured after 8 (filled bars) and 24 h of growth (open bars). Data present average values of two biological replicates with standard deviation.

FIGURE S3 Effect of *sodAB* deletion on amino acid metabolism and ABC transporters. STM WT and STM Δ*sodAB* strains were grown aerobically in LB broth for 18.5 h at 37°C, harvested and proteomes or metabolomes were extracted for quantitative profiling using LC-MS^E^ or GC-MS, respectively. Data represent means of at least three biological replicates for metabolomics or proteomics analyses. Statistical analyses were performed as described for Figure 1.

FIGURE S4 Effect of *acnAB* deletion on amino acid metabolism and ABC transporters. STM WT and STM Δ*acnAB* strains were grown aerobically in LB broth for 18.5 h at 37°C, harvested and proteomes or metabolomes were extracted for quantitative profiling using LC-MS^E^ or GC-MS, respectively. Data represent means of at least three biological replicates for metabolomics or proteomics analyses. Data represent means of at least three biological replicates for the metabolomics or proteomics analyses, respectively. Statistical analyses were performed as described for Figure 1.

FIGURE S5 Increased phagocytic uptake of cytosolic superoxide dismutase-, aconitase-, or fumarase-deficient STM. Phagocytic uptake of STM WT and various mutant strains was determined as for Figure 6. Phagocytosis rates were calculated as percentage of internalized bacteria as fraction of the inoculum. Statistical analyses were performed by Student’s *t*-test and significances are indicated as follows: ^∗∗∗^*p* < 0.001; ^∗∗^*p* < 0.01; ^∗^*p* < 0.05; n.s., not significant.

FIGURE S6 Survival of STM strains after methyl viologen treatment. Various STM strains were cultured o/n in LB at 37°C with aeration. OD_600_ was determined and the cell concentration was adjusted to 6 × 10^5^ bacteria/ml in PBS. The number of viable bacteria/ml was determined by plating serial dilutions on MH agar plates (=inoculum). Methyl viologen was added to a final concentration of 10 mM. After incubation for 2 h at RT, serial dilutions were plated on MH agar plates. Survival rates were calculated as percentage of viable bacteria as fraction of the inoculum. Statistical analyses were performed by Student’s *t*-test and significances are indicated as follows: ^∗^*p* < 0.05; n.s., not significant.

FIGURE S7 Deletion of both aconitases impacts metabolite concentrations and enzyme abundance of central carbon pathways. STM WT and Δ*acnAB* strains were grown aerobically in LB broth for 18.5 h at 37°C. For the proteomic approach, cells were harvested, disrupted with glass beads and proteins precipitated with TCA. After trypsin digest, peptides were analyzed by quantitative LC-MS^E^. Metabolomics analyses were performed as described in Figure 1. For metabolomics analyses, cells were harvested, disrupted, and metabolites were extracted for GC-MS analysis. Heat map colors of oval symbols indicate relative changes in amounts of enzymes detected for STM Δ*acnAB* compared to WT. Heat map colors of square symbols indicate relative changes in amounts of metabolites determined in STM Δ*acnAB* compared to WT. Gray symbols indicate insignificant differences in enzyme or metabolite amounts. Quantitative data are shown for TCA cycle **(A)**, glycolysis **(B)** and pentose phosphate pathway **(C)**. Data represent means of at least four or three biological replicates for the metabolomics or proteomics analyses, respectively. Statistical analyses were performed as described for Figure 1.

TABLE S1 Quantitative proteomics of STM WT and Δs*odAB*.

TABLE S2 GC-MS-based quantification of metabolites of STM WT, Δ*sodAB*, Δ*acnAB*, Δ*fumABC*, and Δ*sdhCDAB*.

TABLE S3 Quantitative proteomics of STM WT and Δ*acnAB*.

TABLE S4 Oligonucleotides and plasmids used in this study.
