# Supplementary figures and images for: Impact of ROS-Induced Damage of TCA Cycle Enzymes on Metabolism and Virulence of Salmonella enterica serovar Typhimurium
Source: Front Microbiol. 2019 Apr 24;10:762. doi: 10.3389/fmicb.2019.00762 (PMC6491894; doi:10.3389/fmicb.2019.00762)

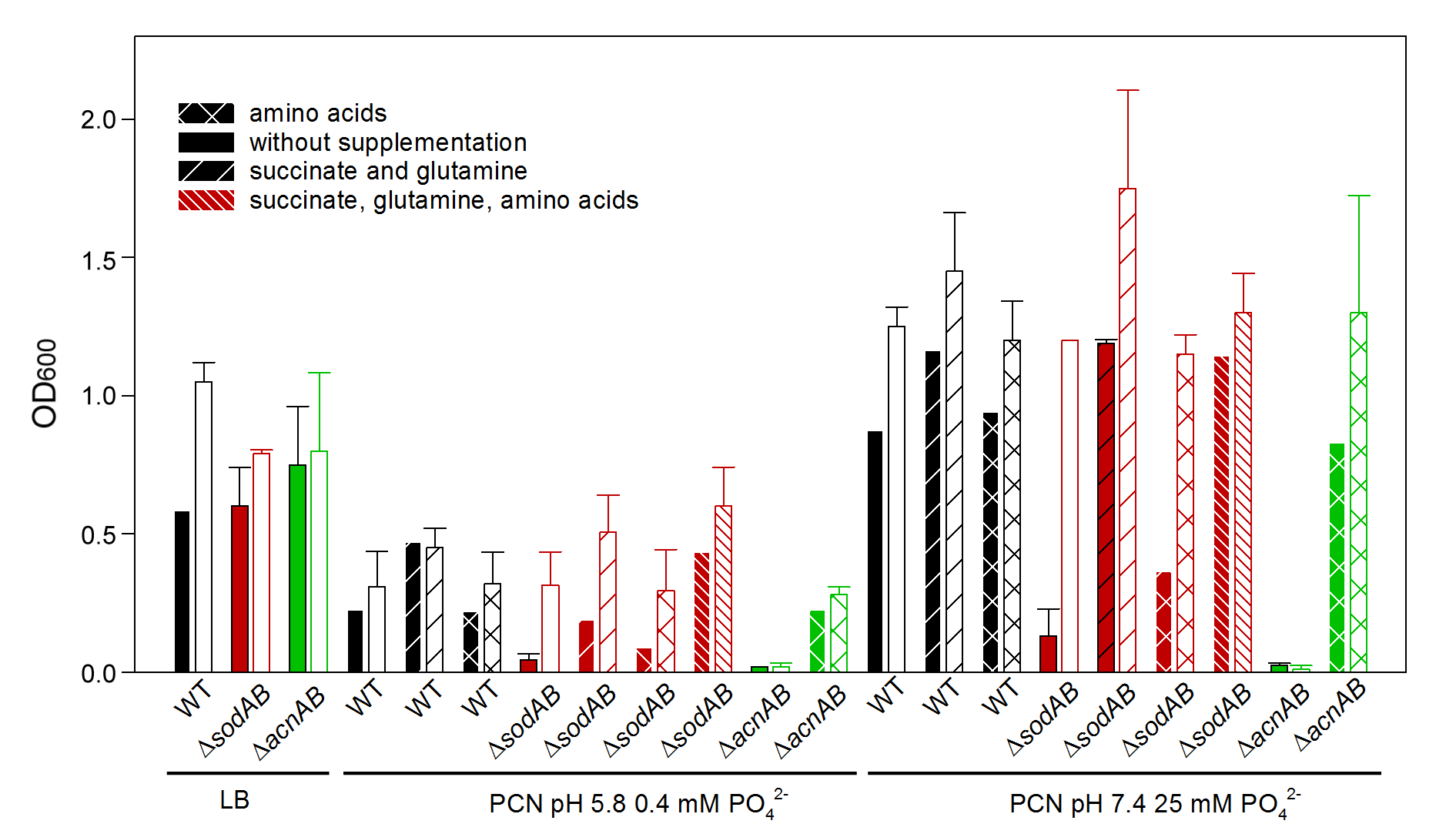

Supplement: FIGURE S2 — Growth of STM WT, ΔsodAB and ΔacnAB under anaerobic conditions. STM strains were cultured o/n in LB broth at 37°C with aeration. Pelleting, washing steps and inoculation was performed as described for Supplementary Figure S1, but using 3 ml culture in 15 ml conical tubes. Cultures were covered with 1 ml paraffin oil, and lids were closed and wrapped with parafilm. Cultures were grown statically at 37°C. Optical density was measured after 8 (filled bars) and 24 h of growth (open bars). Data present average values of two biological replicates with standard deviation. [file Image_2.TIF]

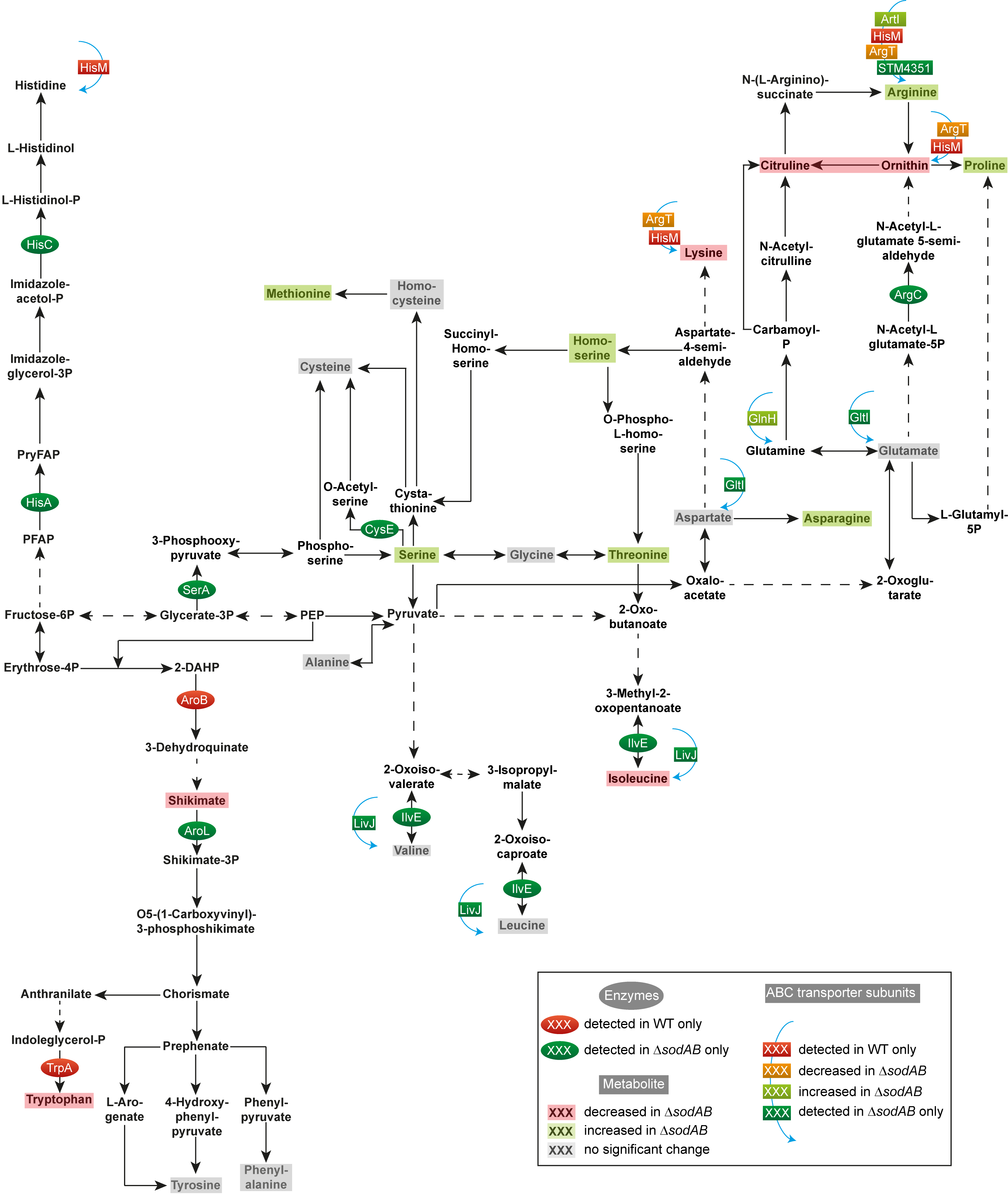

Supplement: FIGURE S3 — Effect of sodAB deletion on amino acid metabolism and ABC transporters. STM WT and STM ΔsodAB strains were grown aerobically in LB broth for 18.5 h at 37°C, harvested and proteomes or metabolomes were extracted for quantitative profiling using LC-MSE or GC-MS, respectively. Data represent means of at least three biological replicates for metabolomics or proteomics analyses. Statistical analyses were performed as described for Figure 1. [file Image_3.TIF]

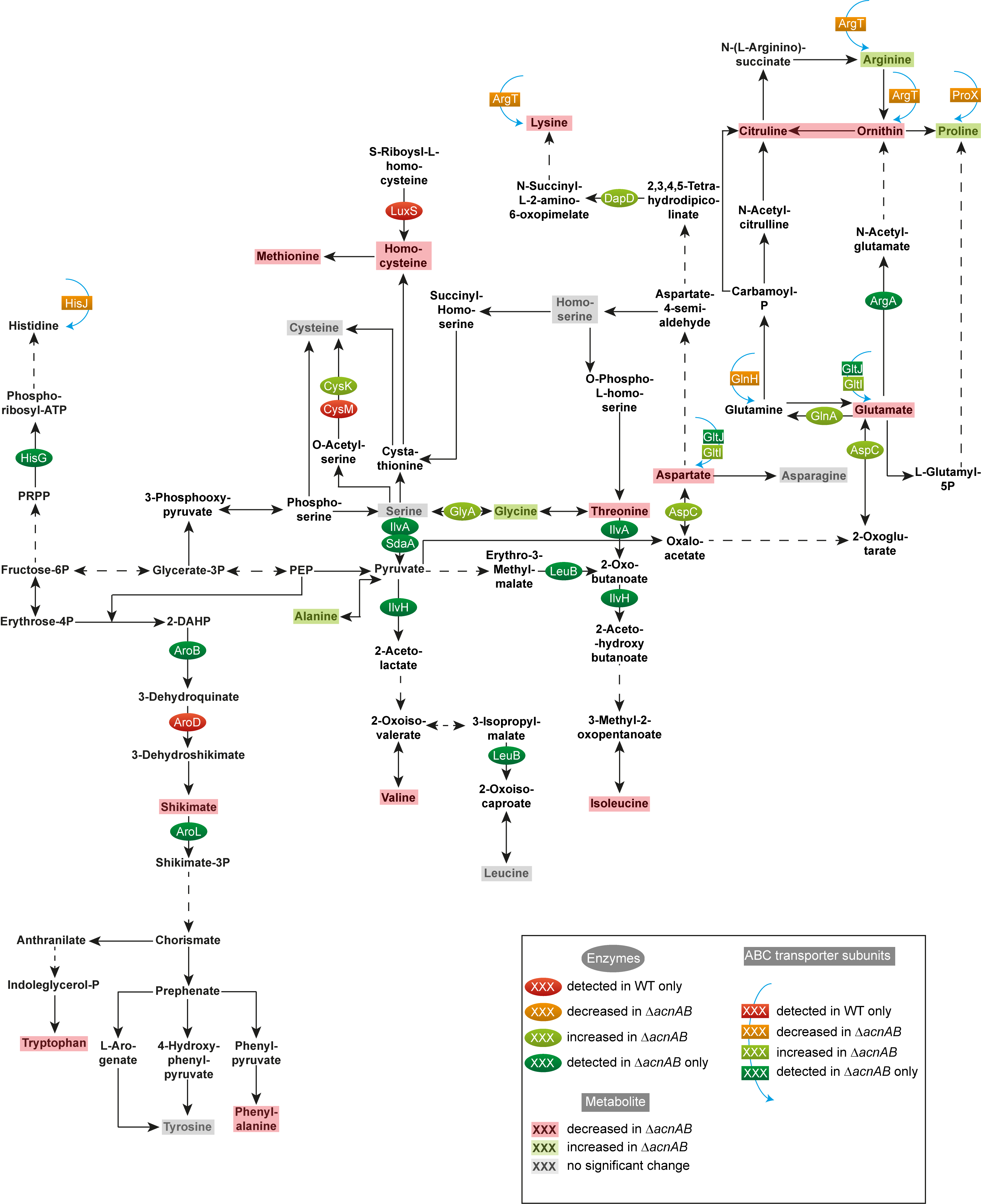

Supplement: FIGURE S4 — Effect of acnAB deletion on amino acid metabolism and ABC transporters. STM WT and STM ΔacnAB strains were grown aerobically in LB broth for 18.5 h at 37°C, harvested and proteomes or metabolomes were extracted for quantitative profiling using LC-MSE or GC-MS, respectively. Data represent means of at least three biological replicates for metabolomics or proteomics analyses. Data represent means of at least three biological replicates for the metabolomics or proteomics analyses, respectively. Statistical analyses were performed as described for Figure 1. [file Image_4.TIF]

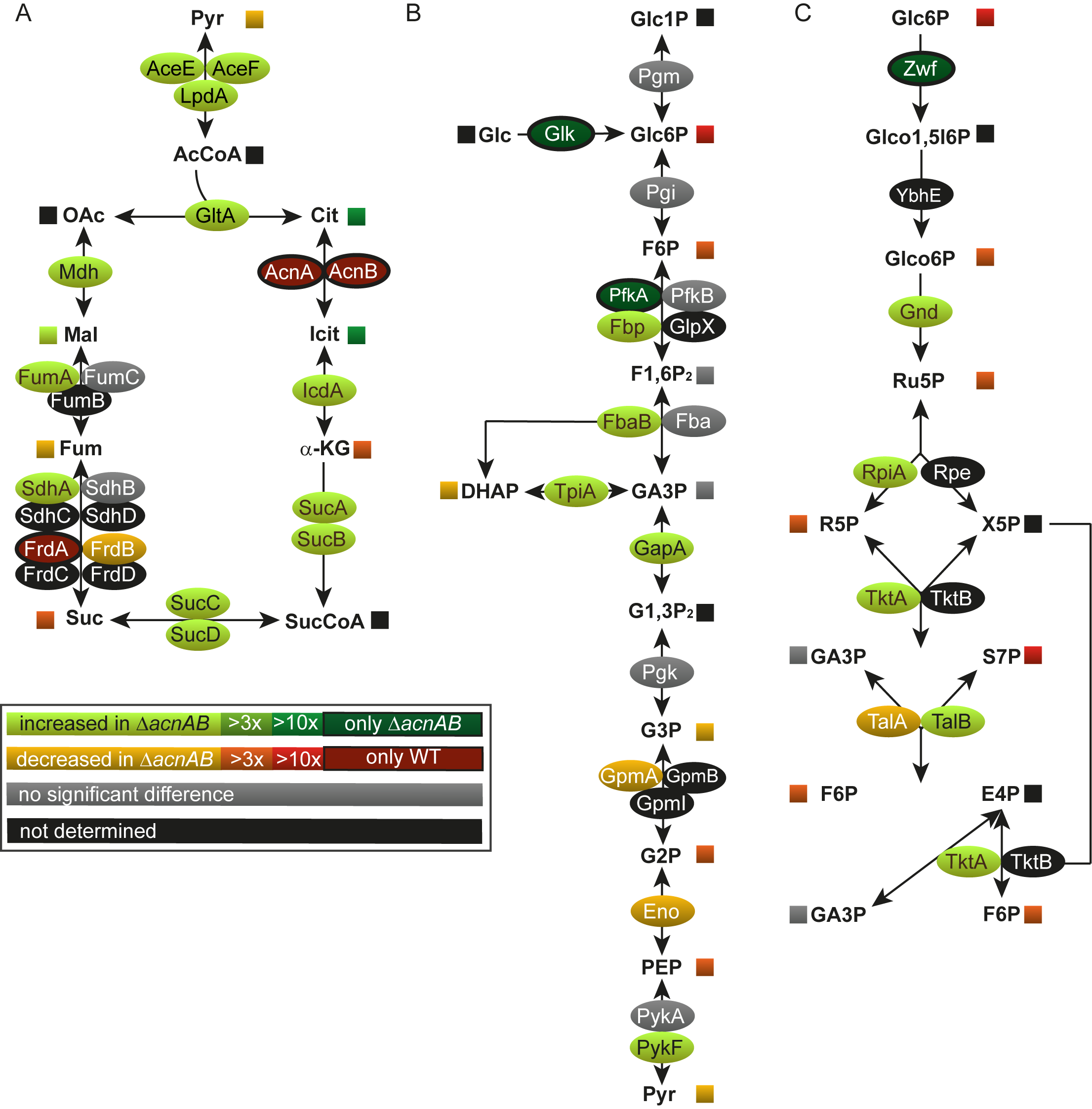

Supplement: FIGURE S7 — Deletion of both aconitases impacts metabolite concentrations and enzyme abundance of central carbon pathways. STM WT and ΔacnAB strains were grown aerobically in LB broth for 18.5 h at 37°C. For the proteomic approach, cells were harvested, disrupted with glass beads and proteins precipitated with TCA. After trypsin digest, peptides were analyzed by quantitative LC-MSE. Metabolomics analyses were performed as described in Figure 1. For metabolomics analyses, cells were harvested, disrupted, and metabolites were extracted for GC-MS analysis. Heat map colors of oval symbols indicate relative changes in amounts of enzymes detected for STM ΔacnAB compared to WT. Heat map colors of square symbols indicate relative changes in amounts of metabolites determined in STM ΔacnAB compared to WT. Gray symbols indicate insignificant differences in enzyme or metabolite amounts. Quantitative data are shown for TCA cycle (A), glycolysis (B) and pentose phosphate pathway (C). Data represent means of at least four or three biological replicates for the metabolomics or proteomics analyses, respectively. Statistical analyses were performed as described for Figure 1. [file Image_7.TIF]
