# Supplementary material for: Impact of ROS-Induced Damage of TCA Cycle Enzymes on Metabolism and Virulence of Salmonella enterica serovar Typhimurium
Source: Front Microbiol. 2019 Apr 24;10:762. doi: 10.3389/fmicb.2019.00762 (PMC6491894; doi:10.3389/fmicb.2019.00762)
Supplement: TABLE S4 — Oligonucleotides and plasmids used in this study. [file Table_4.docx]

**Table S4A. Oligonucleotides used for mutagenesis in this study.**

| Designation | Sequence 5‘-3‘ |
| --- | --- |
| *sodA*-Red-Del13-for | GGCGTTAACACTGTGCCGCTCGACAATAATGGAGATGATTATTCCGGGGATCCGTCGACC |
| *sodA*-Red-Del13-rev | TCGTCCCAGTTCACCACGTTCCAGAACTCTTTGATGTAGTTGTAGGCTGGAGCTGCTTCG |
| *fumA*-Del13-For | GCCCAGAGAATAACCATACCGAGCGGTAAGTGAGAGCACAattccggggatccgtcgacc |
| *fumAC*-Del13-rev2 | ATACTGCCAACCATCAACTCCGGACGTACCCAGGCGTCGAgtaggctggagctgcttcg |
| *fumB*-Del13-for | AGGTTCTTAATACATTTTCTTACTATTAGGCTGGAAGCACATTCCGGGATCCGTCGACC |
| *fumB*-Del13-rev | AACCGATAACGCGGCCCGGGAGAACGCCGGGCCTGCCAGGGTAGGCTGGAGCTGCTTCG |
| *sdhCDAB*-Del13-for | GTCTTAAGGGAATAATAAGAACAGCATGTGGGCGTTATTCATTCCGGGGATCCGTCGACC |
| *sdhCDAB*-Del13-rev | ATAAGACTGTACGTCGCCATCCGGCAACCACTACAACTACGTAGGCTGGAGCTGCTTCG |
| *acnA*-Del13-for | GCGCGGGCCTGCCTGTTTACGGCAGGCCCGATAAACGGCTattccggggatccgtcgacc |
| *acnA*-Del13-rev | AGAATCAGGGTAACGCAACCCTGTCATTTAAGGAGGAGCTgtaggctggagctgcttcg |
| *acnB*-Del13-for | AAGCGCCGCATTATGACAATGAGAGCGAGGAGATATCGTCattccggggatccgtcgacc |
| *acnB*-Del13-rev | ATGAATAAAAAGGGGGCAATTAGCCCCCTTAAAATGTAAAgtaggctggagctgcttcg |
| *ssaV*-Del13-For | TGAGGGAGTCAGGGCGCAACAGTGGCTCAGTGTATGCGCGattccggggatccgtcgacc |
| *ssaV*-Del13-Rev2 | TGTCCGCCAACTCCTCTTCGCTAAGGTCAATACTTTCTACtgtaggctggagctgcttcg |
| *ilvA*-Red-Del13-for | CGAAATTGGGAGGTTGATGATGGCGGAATCTCAACCTCTGATTCCGGGGATCCGTCGACC |
| *ilvA*-Red-Del13-rev | CGAAAAACTAAACGCTGAATTAACCCGCCAGAAAGAACCGTGTAGGCTGGAGCTGCTTCG |
| *trpC*-Red-Del13-for | TTGGGTAGAAGCCCGCAAACAGCAACAGCCGCTGGCCAGTATTCCGGGGATCCGTCGACC |
| *trpC*-Red-Del13-rev | CGCGCAGTGTCTGAAAAACTGAGGCCAGAAGACGAGCATCTGTAGGCTGGAGCTGCTTCG |

**Table S4B. Oligonucleotides used to confirm gene knockouts.**

| Designation | Sequence 5‘-3‘ |
| --- | --- |
| *sodA*-Del-Check-for | GTGTGGAGAGTAAGGCCAAC |
| *sodA*-Del-Check-rev | CATCTGCTGCTCCTTACGCG |
| *fumA*-delcheck13-for | CAGAGAATAACCATACCGAG |
| *fumAC*-delcheck13-rev | CAGCAGAAACAGGTGCAAC |
| *fumB*-delcheck13-for | GCTGGATCTTTGCCGCAATG |
| *fumB*-delcheck13-rev | GTGAGCATGGTCTCTCGTG |
| *sdhCDAB-*delcheck13-for | TCACCTCTGTAACCGAAGTC |
| *sdhCDAB-*delcheck13-rev | CTTGTCTCGCTCTGAACATC |
| *acnA*-delcheck13-for | GCTCATTGTGGGTCGTAATC |
| *acnA*-delcheck13-rev | TCTCGCCTGTTAGGTTTCTC |
| *acnB*-delcheck13-for | GCATTTACCCTACTAACTACTG |
| *acnB*-delcheck13-rev | CCGGTGAGCACAGAATATTG |
| *ssaV*-DelCheck-For | GGGCTTGCAATGAGTTGTTC |
| RT-*ssaN*-rev | TAATAACGCTTCGCCCACGG |
| *trpC*-Del Check-for | GATGCCGCGCATGAGGCCGC |
| *trpC*-Del Check-rev | TGGTTCAGCGCAGGCATCAG |
| *ilvA*-Del Check-for | CAGTTGCAGTTGAGTGAGGC |
| *ilvA*-Del Check-rev | GGATGCTCCGGGCTGGCAGA |
| k1-red-del | CAGTCATAGCCGAATAGCCT |
| k2-red-del | CGGTGCCCTGAATGAACTGC |

## Table S4C. Oligonucleotides used for qPCR

| Designation | Sequence 5‘-3‘ |
| --- | --- |
| 16SrRNA-for3 | CAATTGACGTTACCCGCAGAA |
| 16SrRNA-rev3 | AAGCACCGGCTAACTCCGTGCCA |
| *acnA*-qPCR-for2 | GCGCCGAAGGCCAGATATCC |
| *acnA*-qPCR-rev2 | GGGCCGTGCTTTCCGGTAAC |

## Table S4D. Plasmids used in this study.

| Plasmid | Relevant characteristics | Reference |
| --- | --- | --- |
| pKD46 | Red-expressing vector, ts, Amp^R^ | (Datsenko and Wanner, 2000) |
| pWRG730 | Red-expressing vector, ts, Cm^R^ | (Hoffmann et al., 2017) |
| pKD13 | Template plasmid containing kanamycin cassette, recombinase target sites (FRT), Amp^R^, Kan^R^ | (Datsenko and Wanner, 2000) |
| pE-FLP | flippase-expressing vector, *ts*, Amp^R^ | (St-Pierre et al., 2013) |
| pCP20 | flippase-expressing vector, *ts*, Amp^R^ | (Cherepanov and Wackernagel, 1995) |
